# Heteropolymeric Triplex-Based Genomic Assay® to Detect Pathogens or Single-Nucleotide Polymorphisms in Human Genomic Samples

**DOI:** 10.1371/journal.pone.0000305

**Published:** 2007-03-21

**Authors:** Jasmine I. Daksis, Glen H. Erikson

**Affiliations:** Ingeneus Research, Mississauga, Ontario, Canada; Pasteur Institute, France

## Abstract

Human genomic samples are complex and are considered difficult to assay directly without denaturation or PCR amplification. We report the use of a base-specific heteropolymeric triplex, formed by native duplex genomic target and an oligonucleotide third strand probe, to assay for low copy pathogen genomes present in a sample also containing human genomic duplex DNA, or to assay human genomic duplex DNA for Single Nucleotide Polymorphisms (SNP), without PCR amplification. Wild-type and mutant probes are used to identify triplexes containing *FVL* G1691A, *MTHFR* C677T and *CFTR* mutations. The specific triplex structure forms rapidly at room temperature in solution and may be detected without a separation step. YOYO-1, a fluorescent bis-intercalator, promotes and signals the formation of the specific triplex. Genomic duplexes may be assayed homogeneously with single base pair resolution. The specific triple-stranded structures of the assay may approximate homologous recombination intermediates, which various models suggest may form in either the major or minor groove of the duplex. The bases of the stable duplex target are rendered specifically reactive to the bases of the probe because of the activity of intercalated YOYO-1, which is known to decondense duplex locally 1.3 fold. This may approximate the local decondensation effected by recombination proteins such as RecA *in vivo*. Our assay, while involving triplex formation, is *sui generis*, as it is not homopurine sequence-dependent, as are “canonical triplexes”. Rather, the base pair-specific heteropolymeric triplex of the assay is conformation-dependent. The highly sensitive diagnostic assay we present allows for the direct detection of base sequence in genomic duplex samples, including those containing human genomic duplex DNA, thereby bypassing the inherent problems and cost associated with conventional PCR based diagnostic assays.

## Introduction

Triplex nucleic acids have attracted attention due to their potential regulation of DNA transcription, replication, recombination, chromosome packing, and potential applications in gene therapy [1]–[8]. The “canonical triplex” structures of these studies have consisted of long stretches of uninterrupted homopyrimidine or homopurine strands, typically 12–14 bases in length, that bind in the major groove of duplex DNA, forming Hoogsteen or reverse Hoogsteen hydrogen bonds with bases in the homopurine or purine-rich strand of the duplex. Substantial deviations from strict binding rules are understood to preclude triplex formation [9]–[13]. The utility of such “canonical triplexes” is severely limited due to the requirement of long uninterrupted homopurine sequences in the double-stranded DNA (dsDNA). Such sequence requirements have been understood to preclude the development of triplex-based molecular diagnostics.

The discovery that dsDNA targets, under readily achieved conditions, may specifically bind third-strand DNA oligonucleotides of any base sequence, was accordingly a significant advance [14]. Third-strand binding, facilitated and stabilized by YOYO-1, a cationic bis-intercalator, occurs within 5 minutes at room temperature with great facility, over a wide range of temperatures, under mild and varied incubation conditions and at physiological pH. Specific heteropolymeric triplex formation between non-denatured dsDNA and a ssDNA of mixed base sequence can occur without strand displacement, duplex invasion or the action of recombination proteins, such as recA. This triplex binding capability was used to demonstrate that the Triplex Assay® can accurately detect 1-, 2-, or 3-bp mutations, deletions or insertions in synthetic dsDNA targets and in homozygous or heterozygous polymerase chain reaction (PCR) amplified dsDNA amplicons of varied GC content [14].

PCR amplification based molecular diagnostics are not optimal, due to complexity in optimization, inherent replication error rate, risk of contaminant amplification and cost. The predictable and unpredictable variables that negatively affect PCR amplification based diagnostic methods in real world settings have been recognized as significant [15], [16]. In this report we disclose our advances in SNP and pathogen detection in complex genomic samples using the Triplex Assay® technology. We demonstrate the Genomic Assay®, carried out homogeneously in bulk solution, whereby SNPs or pathogens in samples containing human genomic dsDNA are directly detected by fluorescent signal. We do not have recourse to PCR amplification.

## Results

### Genomic Assay® used to detect pathogens at low copy number

Molecular diagnostic testing for infectious diseases, bacterial and viral, commonly depend upon PCR based methodologies. High copy numbers of the pathogen, such as 10^6^ to 10^8^ colony forming units (CFU)/ml, may be required for successful PCR amplification by diagnostic labs [16]. Use of the Genomic Assay® to detect bacterial genomic dsDNA targets at low concentrations was investigated.

**Figure 1:**
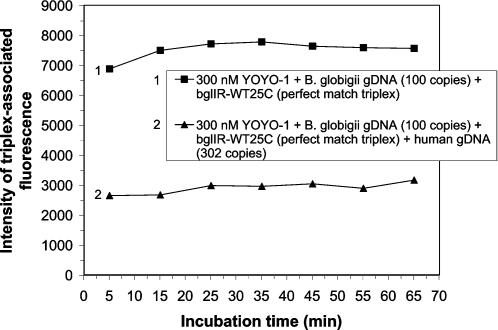
Detection of *Bacillus globigii* genomic dsDNA at low copy number. 100 copies of BG genomic dsDNA (0.46 pg) were reacted with 3.2 pmoles of 25-mer ssDNA probe in the presence of 0.5×TBE, 40 mM TMA-Cl, and 300 nM YOYO-1 in either the presence or absence of 2 ng of human genomic dsDNA (approximately 302 copies). Fluorescent emissions of the reaction mixtures (80 µl) were monitored with the Genexus® Analyzer at a setting of 32% PMT after 5, 15, 25, 35, 45, 55 and 65 minutes of incubation at RT. Intensity of triplex-associated fluorescence is plotted as a function of incubation time for the BG triplexes formed in the presence of either 300 nM YOYO-1 (1) or 300 nM YOYO-1 and human genomic dsDNA (2).

Sequences of the ssDNA probes used are listed in Table 1. Perfectly matched 25-mer triplexes formed in reaction mixtures containing 100 copies of *Bacillus globigii* (BG) genomic dsDNA and probe bglIR-WT25C (probe 1) during a 5 minute incubation in the presence of 300 nM YOYO-1 (Figure 1). All incubations referred to in this report were carried out at room temperature (RT). Duplex DNA employed in our experiments was never subjected to denaturing conditions. The perfect match reaction mixture signal emission intensities were all significantly greater than the combined fluorescence signals emitted by target BG genomic dsDNA and YOYO-1 control intensities and probe and YOYO-1 control intensities (Supplemental Table S1). BG genomic dsDNA controls, which could not be detected at a 32% PMT setting on the Genexus Analyzer, were however detected at the more sensitive 36% PMT setting, consistent with the very low copy number of BG genomic DNA being assayed. To identify triplex-associated signal, all fluorescence emission values from reaction mixtures were corrected for background fluorescence by subtracting the appropriate probe control emission intensity. After initial assay, emissions from all reactions were monitored over time to obtain additional support for the diagnostic conclusions. The efficiency of triplex formation slightly improved during the first 35 minutes of incubation and remained relatively constant thereafter (Figure 1). Other BG genomic DNA samples were prepared as 10-fold serial dilutions, ranging in amount from 10^6^ copies to 10^2^ copies. All were successfully detected using probe 1 and YOYO-1 (data not shown).

**Table 1:** 25-mer and 15-mer ssDNA probe sequences.

Probe Name	Probe No.	Sequence
bglIR-WT25C	1	5′-TATTTTGATTATAGGACATGAAGAT-3′
DR01-WT15	2	5′-GAGCCGAAGGGGCAG-3′
CFTR delta F508-WT25C	3	5′-TAGGAAACACCAAAGATGATATTTT-3′
CFTR delta F508-MUT25C	4	5′-ATAGGAAACACCA---ATGATATTTTCT-3′
CFTR delta I507-WT25C	5	5′-TAGGAAACACCAAAGATGATATTTT-3′
CFTR delta I507-MUT25C	6	5′-ATAGGAAACACCAAAGA---TATTTTCT-3′
CFTR 3659delC-WT25C	7	5′-TGGTTTGGTTGACTTGGTAGGTTTA-3′
CFTR 3659delC-MUT25C	8	5′-ATGGTTTGGTTGACTTG-TAGGTTTA-3′
CFTR 3849+10kbC→T-WT25C	9	5′-GTGTCTTACTCGCCATTTTAATACT-3′
CFTR 3849+10kbC→T-MUT25C	10	5′-GTGTCTTACTC**A**CCATTTTAATACT-3′
CFTR 2789+5G→A-WT25C	11	5′-AATAGGACATGGAATACTCACTTTC-3′
CFTR 2789+5G→A-MUT25C	12	5′-AATAGGACATGGAATA**T**TCACTTTC-3′
CFTR G551D-WT25C	13	5′-ATTCTTGCTCGTTGACCTCCACTCA-3′
CFTR G551D-MUT25C	14	5′-ATTCTTGCTCGTTGA**T**CTCCACTCA-3′
CFTR 621+1G→T-WT25C	15	5′-AAGTATTACCTTCTTATAAATCAAA-3′
CFTR 621+1G→T-MUT25C	16	5′-AAGTATTA**A**CTTCTTATAAATCAAA-3′
CFTR R1162X-WT25C	17	5′-AACTTAAAGACTCGGCTCACAGATC-3′
CFTR R1162X-MUT25C	18	5′-AACTTAAAGACTC**A**GCTCACAGATC-3′
CFTR 1717-1G→A-WT25C	19	5′-TGGAGATGTCCTATTACCAAAAATA-3′
CFTR 1717-1G→A-MUT25C	20	5′-TGGAGATGTC**T**TATTACCAAAAATA-3′
CFTR A455E-WT25C	21	5′-CCAGCAACCGCCAACAACTGTCCTC-3′
CFTR A455E-MUT25C	22	5′-CCAGCAACC**T**CCAACAACTGTCCTC-3′
CFTR G542X-WT25C	23	5′-ATTCCACCTTCTCCAAGAACTATAT-3′
CFTR G542X-MUT25C	24	5′-ATTCCACCTTCTC**A**AAGAACTATAT-3′
CFTR N1303K-WT25C	25	5′-TAGGGATCCAAGTTTTTTCTAAATG-3′
CFTR N1303K-MUT25C	26	5′-TAGGGATCCAA**C**TTTTTTCTAAATG-3′
CFTR R560T-WT25C	27	5′-AGTTATTCACCTTGCTAAAGAAATT-3′
CFTR R560T-MUT25C	28	5′-AGTTATTCAC**G**TTGCTAAAGAAATT-3′
CFTR W1282X-WT25C	29	5′-TTTCCTCCACTGTTGCAAAGTTATT-3′
CFTR W1282X-MUT25C	30	5′-TTTCCT**T**CACTGTTGCAAAGTTATT-3′
MTHFR C677T-WT25C	31	5′-TGATGATGAAATCGGCTCCCGCAGA-3′
MTHFR C677T-MUT25C	32	5′-TGATGATGAAATCG**A**CTCCCGCAGA-3′
FVL G1691A-WT25C	33	5′-CCCTCTGTATTCCTCGCCTGTCCAG-3′
FVL G1691A-MUT25C	34	5′-CCCTCTGTATTCCT**T**GCCTGTCCAG-3′

All 25-mer probes listed were antisense. Probe 2 was a sense 15-mer probe. The site altered in the mutant probes is shown in bold and is underlined. Deletions in the mutant probes are indicated by “dashes”.

Even more remarkable was the ability of the Genomic Assay® to specifically detect 100 copies of bacterial genomic DNA in a sample also containing human genomic dsDNA. Heteropolymeric perfectly matched triplexes were detectably formed between the 100 copies of BG genomic dsDNA and probe 1 in the presence of 300 nM YOYO-1, 40 mM TMA-Cl and 2 ng (302 copies) of human genomic dsDNA (hgDNA) (Figure 1). Moreover, periodic monitoring showed there was a small, progressive increase in triplex-associated fluorescence emission throughout the first 65 minutes of incubation. The fluorescence emissions of the reaction mixtures, whose emissions are shown in Figure 1, were also monitored after 24 hours of incubation. While the BG triplex-associated fluorescence observed in the absence of background hgDNA slightly decreased after 24 hours of incubation, the BG triplex-associated fluorescence observed in the sample also containing hgDNA continued to increase throughout the 24 hours of incubation (Supplemental Table S1). It is noteworthy that the triplex-associated fluorescence emission remained stable over an extended incubation period of 24 hours. The ability to homogeneously assay 0.33 copies of bacterial genomic DNA for each copy of hgDNA present in the sample demonstrates the extreme sensitivity of the Genomic Assay® in detecting pathogens in 80 µl bulk solution.

In this paper we present the use of the Genomic Assay® to detect nucleic acid sequences in various types of samples. Throughout, detection occurs by means of fluorescent emissions from YOYO-1 molecules. The assay may be carried out homogeneously, without a separation step because YOYO-1 molecules, when suitably stimulated, emit with varying intensities dependent upon the nucleic acid structures with which they are complexed and also dependent upon whether certain other reagents are present at very low concentrations. In addition to data in support of these previously unrecognized characteristics of YOYO-1 emissions, we will below, under the “YOYO-1 emissions examined” section of the Results, present data for the unexpected observation that certain lesser concentrations of YOYO-1 will emit much more brightly than greater concentrations of YOYO-1 in the presence of uniform concentrations of nucleic acid oligonucleotides, short duplex DNA or human genomic dsDNA.

### Genomic Assay® identifies 1 bp SNPs in *drosophila* genomic dsDNA

The ability of the Genomic Assay® to identify 1 bp mismatches in non-PCR amplified, non-denatured *drosophila* genomic dsDNA was investigated. The *drosophila* genome was selected as an assay target because its size at 165 million bp is greater than that of an average human chromosome. The mutant *drosophila* genomic dsDNA (ts1 gDNA) contains a 1 bp A to T substitution within the *erg* gene (a seizure locus) [17]. Similar mutations in the human *erg* gene (*HERG*) cause long-QT syndrome, a type of cardiac arrhythmia [18].

**Figure 2 pone-0000305-g002:**
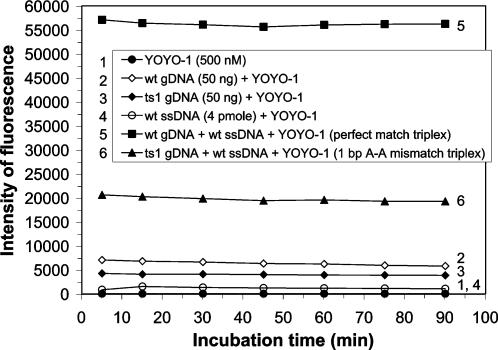
Assay of *drosophila* genomic dsDNA for the ts1 mutation in the *erg* gene. Fifty ng of either wild-type *drosophila* genomic dsDNA (wt gDNA) or mutant *drosophila* genomic dsDNA (ts1 gDNA) were reacted at RT for 5 min with 4 pmoles of wild-type probe 2 in the presence of 0.5×TBE and 500 nM YOYO-1. Fluorescent emissions of the reaction mixtures (100 µl) were monitored with the Genexus® Analyzer at a setting of 30% PMT after 5, 15, 30, 45, 60, 75 and 90 minutes of incubation. The intensity of fluorescence is plotted as a function of incubation time for each sample analyzed. The samples consist of YOYO-1 only with no DNA present (1), wt gDNA with no added ssDNA probe (2), ts1 gDNA with no added ssDNA probe (3), ssDNA probe 2 (4), perfectly matched triplex (5) and 1 bp A–A mismatched triplex (6), as indicated.

The highest triplex-associated fluorescent emission intensity was achieved when wild-type *drosophila* genomic dsDNA (wt gDNA) was reacted with probe 2, indicating that heteropolymeric triplexes, composed of genomic dsDNA bound to a ssDNA probe, can readily form and easily be detected in a homogeneous bulk solution after just 5 min of incubation at RT (Figure 2). The fluorescent intensities attributed to the wt gDNA and YOYO-1 control sample and the wt ssDNA and YOYO-1 control sample were 12% and 1%, respectively, of the total fluorescent intensity emitted from the sample containing perfectly matched triplexes after 5 min of incubation. When the maximum fluorescent emission values were corrected for background fluorescence to account for triplex-associated fluorescent emission, the mismatched dsDNA:ssDNA complexes containing a 1 bp A-A mismatch (ts1 gDNA+probe 2) emitted a triplex-associated fluorescent intensity that was 67% lower than the triplex-associated fluorescent intensity emitted from the perfectly matched dsDNA:ssDNA complexes (wt gDNA+probe 2) after 5 min of incubation (Figure 2). A marginal increase in discrimination between perfectly matched and 1 bp mismatched triplexes was observed upon extended incubation, indicating that the matched triplex complexes formed in the first 5 min, were stable throughout the 90 min incubation period which was periodically monitored. These results demonstrate that the YOYO-1 mediated Genomic Assay® can be employed to assay for 1 bp SNPs in non-denatured genomes of the complexity of *drosophila*, homogeneously in bulk solution.

### Genomic Assay® identifies 1 bp CFTR SNPs and FVL G1691A in human genomic dsDNA

The ability of the Genomic Assay® to identify clinically relevant SNPs in the *CFTR* gene and the factor V gene in non-PCR amplified, non-denatured human genomic dsDNA was investigated. The *CFTR* SNPs were chosen from the 25 most prevalent *CFTR* mutations identified by the American College of Medical Genetics based on data derived from Grody *et al*. [19]. The Factor V Leiden (*FVL*) mutation, G1691A, results in the substitution of glutamine for arginine at position 506 in the amino acid sequence of the coagulation factor V protein [20]. *FVL* G1691A is a SNP responsible for greater than 90 percent of inherited venous thromboembolism attributed to mutations in factor V [21].

Typically the ssDNA probe controls produced higher levels of fluorescent emission in the presence of YOYO-1 as compared to that emitted from the genomic dsDNA target control comprising an identical amount of YOYO-1. On occasion, significantly different fluorescence emission levels were observed for the wild-type and mutant ssDNA probe plus YOYO-1 controls, due to different levels of self hybridization characteristic of various probe sequences in the presence of YOYO-1. Data relevant to these observations are set forth below in the “YOYO-1 emissions examined” section of the Results.

30-mer, 25-mer, 20-mer and 15-mer ssDNA probe sequences were tested for use in the Genomic Assay® (Supplemental Tables S2 and S3). Surprisingly, 25-mer probes were empirically determined to be optimal for maximum discrimination between perfectly matched and 1 bp mismatched triplex-associated fluorescent emissions when hgDNA was being assayed, under the conditions of our testing. To identify triplex-associated signal, fluorescence emission values from the reaction mixtures were corrected for background fluorescence by subtracting the appropriate probe control emission value from the relevant reaction mixture emissions, all emission intensities being acquired after equal periods of incubation.

**Figure 3 pone-0000305-g003:**
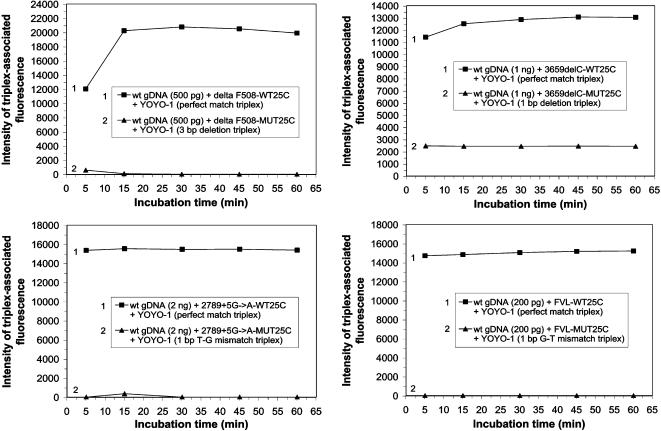
Assay of *CFTR* and *FVL* mutations in human genomic dsDNA. Human genomic dsDNA was extracted from blood (A, B and D) or saliva (C) as described in the text. (A) 500 pg of wild-type human genomic dsDNA (approximately 75 copies) was reacted at RT with 3.2 pmoles of either wild-type or mutant ssDNA probe in the presence of 0.5×TBE and 600 nM YOYO-1. (B–D) 1 ng, 2 ng or 200 pg of wild-type human genomic dsDNA (approximately 151 copies, 302 copies or 30 copies, respectively) was reacted at RT with 3.2 pmoles of either wild-type or mutant ssDNA probe in the presence of 0.5×TBE and 500 nM YOYO-1. (A–D) Reaction mixtures (80 µl) were irradiated as described in the text and analyzed for fluorescent emission. The intensity of triplex-associated fluorescence is plotted as a function of incubation time for each sample analyzed. The samples consist of perfectly matched triplex (1) and mismatched triplex (2) as indicated for *CFTR* delta F508 (A), *CFTR* 3659delC (B), *CFTR* 2789+5G→A (C) and *FVL* G1691A (D).

Heteropolymeric perfectly matched DNA triplexes in reaction mixtures comprised of wild-type hgDNA and wild-type probe delta F508-WT25C (probe 3), formed and were detected after as little as a 5 min incubation in the presence of 600 nM YOYO-1 (Figure 3A and Table 2). It is truly remarkable that perfect match triplex-associated emissions can be significantly greater than the combined emissions of a hgDNA target control with YOYO-1 and a probe control with YOYO-1 (Table 2). The percent variation of triplex-associated fluorescence (TAF) signals was 0.2–5.0% when triplicate samples prepared at the same time were assayed on the same plate concurrently. The signal of perfect match triplex formation increased dramatically during the first 15 min of incubation, after which a plateau in fluorescence emission level was observed (Figure 3A). The triplex-associated fluorescent emissions produced by a 3 bp deletion triplex in reaction mixtures comprising either 500 pg or 250 pg hgDNA and mutant probe delta F508-MUT25C (probe 4) were 95% and 100% lower, respectively, than those emitted by the perfectly matched triplexes comprising comparable amounts of hgDNA and probe 4 after 5 min of incubation, and remained at this low level throughout the 75 min incubation period monitored (Figure 3A and Table 2).

**Table 2:** Triplex assays of human genomic dsDNA for *CFTR* delta F508 (3 bp AAG deletion).

Sample	Fluorescence on Genexus argon laser @ PMT 34 after 5 min	TAF	% of difference relative to perfect match TAF	Fluorescence on Genexus argon laser @ PMT 34 after 15 min	TAF	% of difference relative to perfect match TAF
1) YOYO-1 (600 nM)	0			0		
2) delta F508-WT25C (3.2 pmole) (antisense)	7650			6326		
3) delta F508-MUT25C (3.2 pmole) (antisense)	7626			7123		
4) wt gDNA (500 pg)	2985			3041		
5) wt gDNA (500 pg)+delta F508-WT25C (perfect)	19688	12038		26578	20252	
6) wt gDNA (500 pg)+delta F508-MUT25C (3 bp AAG del)	8211	585	−95.1	7211	88	−99.5
7) wt gDNA (250 pg)	1110			1097		
8) wt gDNA (250 pg)+delta F508-WT25C (perfect)	12176	4526		11348	5022	
9) wt gDNA (250 pg)+delta F508-MUT25C (3 bp AAG del)	7097	<0	−100	6399	<0	−100
**Sample**	**Fluorescence on Genexus argon laser @ PMT 34 after 30 min**	**TAF**	**% of difference relative to perfect match TAF**	**Fluorescence on Genexus argon laser @ PMT 34 after 45 min**	**TAF**	**% of difference relative to perfect match TAF**
1) YOYO-1 (600 nM)	0			0		
2) delta F508-WT25C (3.2 pmole) (antisense)	5716			5555		
3) delta F508-MUT25C (3.2 pmole) (antisense)	7135			7167		
4) wt gDNA (500 pg)	3035			2977		
5) wt gDNA (500 pg)+delta F508-WT25C (perfect)	26488	20772		26065	20510	
6) wt gDNA (500 pg)+delta F508-MUT25C (3 bp AAG del)	6935	<0	−100	6744	<0	−100
7) wt gDNA (250 pg)	1064			1096		
8) wt gDNA (250 pg)+delta F508-WT25C (perfect)	11525	5809		11142	5587	
9) wt gDNA (250 pg)+delta F508-MUT25C (3 bp AAG del)	6151	<0	−100	6008	<0	−100
**Sample**	**Fluorescence on Genexus argon laser @ PMT 34 after 60 min**	**TAF**	**% of difference relative to perfect match TAF**	**Fluorescence on Genexus argon laser @ PMT 34 after 75 min**	**TAF**	**% of difference relative to perfect match TAF**
1) YOYO-1 (600 nM)	0			0		
2) delta F508-WT25C (3.2 pmole) (antisense)	5147			4943		
3) delta F508-MUT25C (3.2 pmole) (antisense)	6978			7097		
4) wt gDNA (500 pg)	2912			2831		
5) wt gDNA (500 pg)+delta F508-WT25C (perfect)	25064	19917		25091	20148	
6) wt gDNA (500 pg)+delta F508-MUT25C (3 bp AAG del)	6402	<0	−100	6180	<0	−100
7) wt gDNA (250 pg)	1013			989		
8) wt gDNA (250 pg)+delta F508-WT25C (perfect)	10407	5260		9974	5031	
9) wt gDNA (250 pg)+delta F508-MUT25C (3 bp AAG del)	5723	<0	−100	5531	<0	−100

The target was human genomic dsDNA, wild-type for *CFTR*. The 25-mer probes were delta F508-WT25C (wild-type) and delta F508-MUT25C (mutant). 600 nM YOYO-1 was present in each sample. TAF indicates Triplex-Associated Fluorescence.

When 1 ng of wild-type hgDNA was reacted with wild-type probe 3659delC-WT25C (probe 7) in the presence of 500 nM YOYO-1, triplex-associated signals were detected after 5 min of incubation (Figure 3B). A slight increase in triplex-associated fluorescence was observed over the 60 min of incubation. The triplex-associated fluorescent emissions from a 1 bp deletion triplex in a reaction mixture comprised of 1 ng hgDNA and mutant probe 3659delC-MUT25C (probe 8) remained constant throughout the 60 min incubation period and were 81% lower than those emitted from the reaction mixture containing the perfectly matched triplexes (Figure 3B).

The highest fluorescent emission intensity was achieved when wild-type hgDNA was reacted with wild-type probe 2789+5G→A-WT25C (probe 11), indicating perfect match triplex formation after 5 min of incubation in the presence of 500 nM YOYO-1 (Figure 3C and Table 3). The emissions from these perfectly matched DNA triplexes remained stable throughout the 60 min incubation period. When 2 ng of hgDNA was reacted with mutant probe 2789+5G→A-MUT25C (probe 12) to form a 1 bp T–G mismatch triplex, the triplex-associated fluorescent emission was 100% lower than that emitted by the reaction mixture containing the perfectly matched triplex after 5 min incubation and remained constant thereafter (Figure 3C and Table 3). In this instance the fluorescent emissions from reaction mixtures containing mismatched genomic DNA triplexes were less than the emissions from the comparable probe and YOYO-1 control. We hypothesize that this result occurs because, while YOYO-1 facilitates and signals self-association of the 25-mer probes when no genomic dsDNA is present, such self-associated probe complexes tend to disperse, along with any complexed YOYO-1, upon introduction of the genomic target and that only a small offsetting gain in fluorescence occurs when YOYO-1 intercalates into the duplex genomic target or into imperfect triple stranded structures. When emissions from samples containing mismatched triplexes are less intense than emissions from the relevant probe and YOYO-1 control, very desirable assay conditions have been achieved.

**Table 3 pone-0000305-t003:** Triplex assays of human genomic dsDNA (purified from saliva) for *CFTR* 2789+5G→A (1 bp T–G mismatch).

Sample	Fluorescence on Genexus argon laser @ PMT 30 after 5 min	TAF	% of difference relative to perfect match TAF	Fluorescence on Genexus argon laser @ PMT 30 after 15 min	TAF	% of difference relative to perfect match TAF
1) YOYO-1 (500 nM)	0			0		
2) 2789+5G→A-WT25C (3.2 pmole) (antisense)	23291			22608		
3) 2789+5G→A-MUT25C (3.2 pmole) (antisense)	22804			22071		
4) wt gDNA (4 ng)	1530			1856		
5) wt gDNA (4 ng)+2789+5G→A-WT25C (perfect)	44651	21360		43624	21016	
6) wt gDNA (4 ng)+2789+5G→A-MUT25C (1 bp T-G)	23893	1089	−94.9	23531	1460	−93.1
7) wt gDNA (2 ng)	21			19		
8) wt gDNA (2 ng)+2789+5G→A-WT25 (perfect)	38650	15359		38142	15534	
9) wt gDNA (2 ng)+2789+5G→A-MUT25 (1 bp T–G)	22453	<0	−100	22430	359	−97.7
**Sample**	**Fluorescence on Genexus argon laser @ PMT 30 after 30 min**	**TAF**	**% of difference relative to perfect match TAF**	**Fluorescence on Genexus argon laser @ PMT 30 after 45 min**	**TAF**	**% of difference relative to perfect match TAF**
1) YOYO-1 (500 nM)	0			0		
2) 2789+5G→A-WT25C (3.2 pmole) (antisense)	22243			21877		
3) 2789+5G→A-MUT25C (3.2 pmole) (antisense)	21847			21714		
4) wt gDNA (4 ng)	1959			2102		
5) wt gDNA (4 ng)+2789+5G→A-WT25C (perfect)	42950	20707		42412	20535	
6) wt gDNA (4 ng)+2789+5G→A-MUT25C (1 bp T–G)	23077	1230	−94.1	22806	1092	−93.8
7) wt gDNA (2 ng)	18			18		
8) wt gDNA (2 ng)+2789+5G→A-WT25 (perfect)	37707	15464		37351	15474	
9) wt gDNA (2 ng)+2789+5G→A-MUT25 (1 bp T–G)	21835	<0	−100	21541	<0	−100

The target was human genomic dsDNA, wild-type for *CFTR*. The 25-mer probes were 2789+5G→A-WT25C (wild-type) and 2789+5G→A-MUT25C (mutant). 500 nM YOYO-1 was present in each sample. TAF indicates Triplex-Associated Fluorescence.

Heteropolymeric perfectly matched triplexes were detected in reaction mixtures containing either 2 ng, 1 ng, 500 pg or 200 pg hgDNA and probe FVL G1691A-WT25C (probe 33), formed during a 5 min incubation in the presence of 500 nM YOYO-1 (Figure 3D and Supplemental Table S4). The perfect match triplex signal levels were all significantly greater than the combined fluorescence signals of target amounts of hgDNA with YOYO-1 control and wild-type probe with YOYO-1 control (Supplemental Table S4). As was usual, the triplex-associated emissions from the reaction mixtures were stable throughout the 60 min incubation period. The triplex-associated fluorescent emissions from reaction mixtures containing between 2 ng and 200 pg of genomic target and probe FVL G1691A-MUT25C (probe 34) and producing 1 bp G–T mismatched triplexes were consistently less than probe 34 plus YOYO-1 control values after 5, 15, 30, 45 and 60 minute incubations (Supplemental Table S4). Therefore the reactions produced no detected triplex-associated fluorescence. Comparable results were observed when 500 pg, 200 pg, 100 pg or 75 pg of hgDNA were reacted with 3.2 pmoles of either wild-type probe CFTR 2789+5G→A-WT25C (probe 11) or mutant probe CFTR 2789+5G→A-MUT25C (probe 12) (Supplemental Table S5).

These results collectively demonstrate the high level of efficiency and specificity of the Genomic Assay® when practised in bulk solution with 25-mer ssDNA probes and YOYO-1, to directly detect mismatches between probes and non-denatured, non-amplified human genomic dsDNA targets, over a broad range of genomic target concentrations. SNPs in human genomic targets ranging from 4 ng to 75 pg, approximately 604 copies to 11 copies, were identified in the Genomic Assay® using varied YOYO-1 concentrations, homogeneously and in an 80 µl solution.

The human genomic DNA samples assayed in Figures 3A, 3B and 3D were purified from blood. We assayed for the CFTR 2789+5G→A mutation in both human genomic dsDNA purified from blood (Supplemental Table S6) and purified from treated saliva/sputum samples (Figure 3C and Table 3), which had been stored at RT for 1 week following incubation at 50°C for 30 min. The 50°C incubation is recommended by the manufacturer of the Oragene oral sample acquisition kit to allow long-term storage at RT. In side by side comparisons, triplex-associated fluorescent emissions from the reaction mixtures containing hgDNA purified from blood or from treated saliva/sputum, indicated that perfectly matched triplexes formed with equivalent efficiency after 5 min incubation in the presence of 500 nM YOYO-1 (Table 3 and Supplemental Table S6). The triplex-associated emissions remained stable throughout a 60 min incubation during which they were monitored. The triplex-associated fluorescent emissions from reaction mixtures containing 1 bp T-G mismatched triplexes were very low, regardless of the origin of the sample or which of the two methods of purification of the sample had been employed (Table 3 and Supplemental Table S6).

This demonstrates that the Genomic Assay® of hgDNA purified from saliva/sputum in the Oragene DNA collection kit can be as efficient and specific as one in which the hgDNA is purified from blood. Purification of genomic DNA, as occurs when using Qiagen kits, is therefore not a requirement for the Genomic Assay® of samples containing hgDNA. The acquisition of hgDNA for molecular assay from oral samples offers clear advantages in cost, handling, storing and shipping over acquisition of samples from blood. It would also be greatly preferred by sample donors. It therefore opens the way for convenient point of care testing employing the Genomic Assay®. In side by side comparisons, 1 ml of peripheral blood, purified by Qiagen kit, yielded more than enough human genomic DNA to perform over 9,000 assays as described herein, if 1 ng were assayed per reaction. A typical saliva/sputum sample, purified with an Oragene kit, produced enough human genomic DNA for over 7,000 assays, if 1 ng were needed per reaction.

### Genomic Assay® identifies MTHFR C677T in homozygous and heterozygous human genomic dsDNA samples

The ability of the Genomic Assay® to identify the methylenetetrahydrofolate reductase (*MTHFR*) mutation, C677T, in non-PCR amplified, non-denatured, human genomic dsDNA was investigated. The *MTHFR* 677 polymorphism is a cytosine to thymine substitution in the sense strand of the *MTHFR* gene, resulting in an alanine to valine substitution in the MTHFR enzyme [22].

Different fluorescence emission levels were observed for the wild-type probe MTHFR C677T-WT25C (probe 31) plus YOYO-1 control and the mutant probe MTHFR C677T-MUT25C (probe 32) plus YOYO-1 control, due to differences in levels of self hybridization which are characteristic of each probe sequence in the presence of YOYO-1 (Supplemental Tables S7 and S8). To identify triplex-associated signal, fluorescence emission values from the reaction mixtures, containing hgDNA, probe and YOYO-1, were corrected for background fluorescence by subtracting the appropriate probe control emission value from the relevant reaction mixture emission, both of which were measured after the same duration of incubation.

**Figure 4 pone-0000305-g004:**
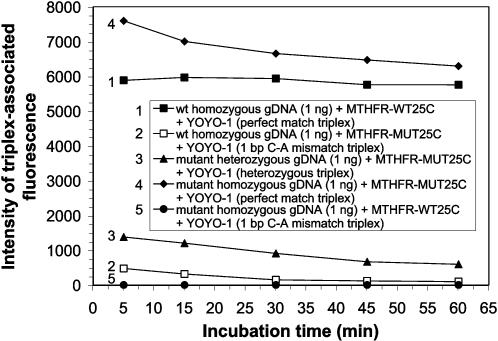
Assay of *MTHFR* C677T in homozygous and heterozygous human genomic dsDNA samples. Human genomic dsDNA that was either wild-type homozygous, mutant heterozygous or mutant homozygous with respect to *MTHFR* C677T, was extracted from blood as described in the text. One ng of human genomic dsDNA (approximately 151 copies) was reacted at RT with 3.2 pmoles of either wild-type or mutant ssDNA probe in the presence of 0.5×TBE and 500 nM YOYO-1. Reaction mixtures (80 µl) were irradiated as described in the text and analyzed for fluorescent emission. The intensity of triplex-associated fluorescence is plotted as a function of incubation time for each sample analyzed. The samples consist of perfectly matched triplexes (1 and 4), mutant heterozygous triplexes (3) and mismatched triplexes (2 and 5) as indicated for *MTHFR* C677T.

Perfectly matched DNA triplexes in reaction mixtures comprised of either wild-type homozygous hgDNA and wild-type probe MTHFR C677T-WT25C (probe 31) or mutant homozygous hgDNA and mutant probe MTHFR C677T-MUT25C (probe 32), formed efficiently and were detected after just 5 min of incubation in the presence of 500 nM YOYO-1 (samples 1 and 4, respectively, in Figure 4). The efficiency of matched triplex formation, signaled by the appearance of triplex-associated fluorescence, was slightly greater in the reaction mixtures comprising 1 ng mutant homozygous hgDNA and mutant probe 32, than that comprising 1 ng wild-type homozygous hgDNA and wild-type probe 31, after 5 min of incubation. The triplex-associated fluorescent emission intensities observed from reaction mixtures containing either of the two perfectly matched triplexes were more similar when 2 ng, instead of 1 ng, of hgDNA was present in the 80 µl bulk solution reaction mixtures (Supplemental Tables S7 and S8).

When 1 ng mutant heterozygous hgDNA was reacted with mutant probe 32 (sample 3, Figure 4), the triplex-associated fluorescent emissions were 77% less after 5 min incubation and 90% less after 60 min incubation, than those emitted from the reaction mixture containing the perfectly matched triplexes comprised of 1 ng wild-type homozygous hgDNA and wild-type probe 31 (sample 1, Figure 4), each evaluated after comparable periods of incubation.

The triplex-associated fluorescent emissions from 1 bp C–A mismatch triplexes in a reaction mixture comprised of 1 ng wild-type homozygous hgDNA and mutant probe 32 (sample 2, Figure 4) were 92% less after 5 min and 98% less after 60 min incubation, than those emitted from the reaction mixture containing the perfectly matched triplexes comprised of 1 ng wild-type homozygous hgDNA and wild-type probe 31 (sample 1, Figure 4), each evaluated after comparable periods of incubation. The triplex-associated fluorescent emissions from 1 bp C–A mismatch triplexes in a reaction mixture comprised of 1 ng mutant homozygous hgDNA and wild-type probe 31 (sample 5, Figure 4) were consistently less than the wild-type probe 31 plus YOYO-1 control value. Therefore the reaction produced no detected triplex-associated fluorescence.

These results demonstrate the high efficiency and specificity of the Genomic Assay® in assaying for SNPs in wild-type homozygous, mutant heterozygous or mutant homozygous hgDNA samples homogeneously and in bulk solution. The results shown were obtained using antisense 25-mer wild-type and mutant probes for *MTHFR* C677T. Comparable results were observed when analogous reactions were performed using sense 25-mer wild-type and mutant probes for *MTHFR* C677T (data not shown). The sensitivity of the Genomic Assay® to assay for *MTHFR* C677T was further demonstrated when human genomic dsDNA targets, ranging in weight from 2 ng to 200 pg, were successfully assayed with wild-type probe 31 and mutant probe 32 in a final reaction volume of 80 µl (Supplemental Table S9).

Numerous SNPs, in regions having widely disparate GC contents, have been successfully and repeatedly assayed in hgDNA using the Genomic Assay® (Table 4). On occasion, variations in reagent concentrations or protocol were advantageous. The triplex-associated fluorescent emissions observed from the mismatched triplexes varied between 100% to 54% lower than those observed from the respective perfectly matched triplexes after 5 min of incubation (Table 4).

**Table 4:** Data from Assays of Human Genomic DNA Samples.

Mutation	Source	Number of tests	Percentage GC in probe sequence	Percentage difference of mismatched TAF relative to perfect match TAF
*CFTR* delta F508	blood	102	28%	−100% to −81%
*CFTR* delta I507	blood	6	28%	−100% to −85%
*CFTR* 3659delC	blood	11	40%	−100% to −55%
*CFTR* 3849+10kbC→T	blood	9	36%	−100% to −82%
*CFTR* 2789+5G→A	blood	16	36%	−100% to −75%
*CFTR* 2789+5G→A	saliva	13	36%	−100% to −66%
*CFTR* G551D	blood	11	48%	−100% to −61%
*CFTR* 621+1G→T	blood	5	20%	−100% to −57%
*CFTR* R1162X	blood	6	44%	−67% to −36%
*CFTR* 1717-1G→A	blood	12	32%	−100% to −58%
*CFTR* A455E	blood	9	60%	−100% to −89%
*CFTR* G542X	blood	6	36%	−100% to −60%
*CFTR* N1303K	blood	8	32%	−100% to −83%
*CFTR* R560T	blood	6	28%	−100% to −54%
*CFTR* W1282X	blood	14	36%	−100% to −74%
*MTHFR* C677T	blood	55	52%	−100% to −72%
*FVL* G1691A	blood	34	60%	−100% to −81%

TAF indicates Triplex-Associated Fluorescence.

### The Genomic Assay® can employ the waxing and/or waning of triplex-associated fluorescent emissions

**Figure 5:**
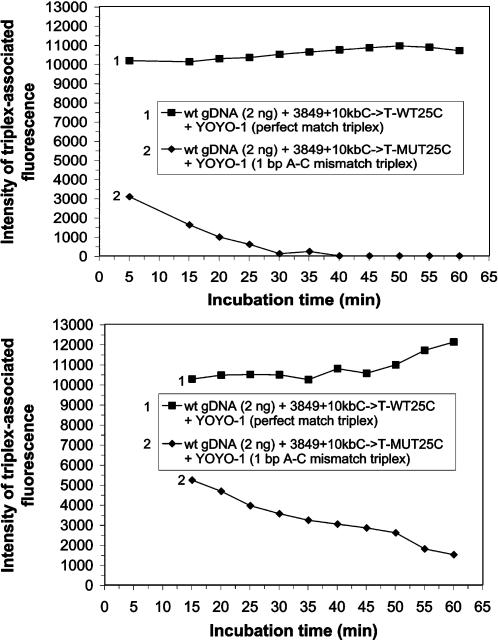
Waxing and waning of triplex-associated fluorescent emissions can enhance a homogeneous in solution assay. (A, B) Human genomic dsDNA was extracted from blood as described in the text. Two ng of wild-type human genomic dsDNA (approximately 302 copies) was reacted at RT with 3.2 pmoles of either wild-type or mutant ssDNA probe in the presence of 0.5×TBE, 40 mM TMA-Cl and 500 nM YOYO-1. (A) YOYO-1 was added last to the reaction mixtures. (B) Human genomic dsDNA was added last to the reaction mixtures. (A, B) Reaction mixtures (80 µl) were irradiated as described in the text and analyzed for fluorescent emission. The intensity of triplex-associated fluorescence is plotted as a function of incubation time for each sample analyzed. The samples consist of perfectly matched triplex (1) and mismatched triplex (2) as indicated for *CFTR* 3849+10kbC→T.

The effect of addition of various kosmotropic agents to the reaction mixture when assaying to identify various SNPs in human genomic dsDNA was investigated. In the presence of 40 mM of the kosmotropic cation, tetramethylammonium chloride (TMA-Cl), the fluorescent emissions from the wild-type probe CFTR 3849+10kbC→T-WT25C (probe 9) and mutant probe CFTR 3849+10kbC→T-MUT25C (probe 10) controls were very similar (Supplemental Table S10). Duplicate triplex reaction mixtures were assembled. In one instance YOYO-1 was added last, with the mixture incubated for 5 min before irradiation (Figure 5A). In the other instance all components of the reaction mixture (except hgDNA) had been mixed, incubated for 5 min and irradiated once before hgDNA was added, followed by a further 5 min incubation and another irradiation (Figure 5B). To identify triplex-associated emissions, fluorescence emission values from the reaction mixtures were corrected for background fluorescence by subtracting the appropriate probe control emission values from the relevant reaction mixture emissions, all of which were acquired after comparable periods of incubation.

The triplex-associated fluorescent emissions from perfectly matched triplexes in reaction mixtures consisting of hgDNA and wild-type probe 3849+10kbC→T-WT25C (probe 9), monitored after a 15 min incubation in the presence of 40 mM TMA-Cl and 500 nM YOYO-1, were similar, whether YOYO-1 or hgDNA had been added last to the reaction mixtures (Figures 5A and 5B). Under both protocols, the perfectly matched triplex-associated fluorescent emissions increased when monitored over the first 60 min of incubation.

In the reaction mixture containing 40 mM TMA-Cl, to which YOYO-1 had been added last, the triplex-associated emissions from 1 bp A–C mismatched triplexes, consisting of hgDNA and mutant probe 3849+10kbC→T-MUT25C (probe 10), were 70% less after 5 min of incubation and were 100% less after 30 min of incubation, than those emitted from a similarly monitored reaction mixture containing the perfectly matched triplexes (Figure 5A). The triplex-associated emissions from the 1 bp A–C mismatched triplexes in these reaction mixtures remained constant between 30 min and 60 min of incubation. In the reaction mixture containing 40 mM TMA-Cl, to which hgDNA had been added last, the triplex-associated emissions from 1 bp A–C mismatched triplexes consisting of hgDNA and mutant probe 3849+10kbC→T-MUT25C (probe 10) were 49% less after 15 min of incubation and were 88% less after 60 min of incubation, than those emitted from a similarly monitored reaction mixture containing the perfectly matched triplexes (Figure 5B).

These results demonstrate that the addition of 40 mM TMA-Cl to the triplex reaction mixture can result in an enhanced progressive increase in triplex-associated fluorescent emission levels from perfectly matched triplexes and an enhanced progressive decrease in triplex-associated fluorescent emission levels from mismatched triplexes, which pattern of emission changes we refer to as “waxing and waning”. Increases in discrimination levels between perfect match binding and mismatch binding can be observed over time. A pronounced waxing and waning emission pattern was also observed in Genomic Assays® of hgDNA samples for *CFTR* 2789+5G→A, *CFTR* delta F508 or *FVL* G1691A detection when 45 mM TMA-Cl, 40 mM TMA-Cl or both 50 mM TMA-Cl and 20 mM NaCl were included, respectively, in the reaction mixtures containing 500 nM YOYO-1 (data not shown).

Numerous kosmotropic cations have been used by us in conjuction with YOYO-1 to generally improve the specificity of the Genomic Assay® and to enhance the waxing and waning of triplex-associated emissions during SNP detection (data not shown). These include 50 to 80 mM NaCl, 10 to 60 mM Na_2_SO_4_, 50 mM Na_2_HPO_4_, 125 to 250 mM (NH_4_)_2_SO_4_, 30 mM TriMA-Cl, 30 to 52.5 mM TMA-Cl, each added separately, or 50 mM TMA-Cl in combination with 10 to 20 mM NaCl. The benefits of adding one or more kosmotropic cations to a Genomic Assay® reaction mixture and the best concentrations for addition under any set of selected assay conditions must be determined experimentally.

### YOYO-1 emissions examined

YOYO-1 will intercalate into a variety of multi-stranded nucleic acid complexes. It is fluorescent when intercalated and suitably irradiated. In the Genomic Assay® we used it to promote and signal the formation of heteropolymeric, specific triplex useful in diagnostics and analysis. As shown throughout this paper, the emissions from YOYO-1 complexed in triple stranded structures were detectable over and above the emissions generated by YOYO-1 complexed with genomic duplex DNA present in the reaction mixture and very significant emissions by YOYO-1 complexed with probe:probe binding. Our triplex assay reaction mixtures typically contained concentrations of between 300 to 600 nM YOYO-1 when assaying genomic samples. We conclude that YOYO-1 allowed for such assaying because it did not produce a constant emission per intercalated molecule. Triplex intercalated YOYO-1's fluoresced much brighter than did duplex intercalated YOYO-1's. We also observed that YOYO-1 molecules were much brighter when intercalated into complexes of identical short oligonucleotides, such as the probes used in our assay, than when a like number of YOYO-1 molecules were in the presence of genomic duplex DNA.

Probe:probe associated fluorescence constituted the major source of unwanted background signal in our YOYO-1 promoted triplex assay conducted homogeneously in solution. Such probe:probe complex emissions were quantified in the “probe plus YOYO-1” control which accompanied each triplex assay. Emissions from probe:probe complexes must be determined individually, as each probe base sequence has a characteristic ability to not only form hairpin structures and self-dimer hybridized complexes, but also to form parallel homologous structures stabilized and signalled by intercalated YOYO-1 molecules (Daksis and Erikson, unpublished observations). YOYO-1's non-specific intercalation into duplex genomic DNA also produced unwanted background signal in our assay, albeit of a vastly lesser intensity than that emitted from YOYO-1s intercalated into probe:probe complexes. This observation was unexpected, but consistently observed. We investigated such background emissions by having a “duplex target plus YOYO-1” control which accompanied each triplex assay. Fortunately, the signal emitted from triple-stranded nucleic acids and complexed YOYO-1 molecules was so intense, that the triple-stranded structure could be readily detected homogeneously in solution. When genomic dsDNA was added last to a reaction mixture, we believe probe:probe complexes dissolved and intercalated YOYO-1 molecules were released.

To demonstrate YOYO-1's ability to promote and signal the formation of probe:probe complexes, we monitored fluorescent emissions from 80 µl final volume hybridization mixtures containing 0.5×TBE, pH 8.3, 40 mM TMA-Cl, 3.2 pmole of 25-mer heteropolymeric DNA oligo probe bglIR-WT25C (probe 1) and various concentrations of YOYO-1, ranging from 50 nM to 1000 nM, at 50 nM intervals. There was no emission from YOYO-1 over this range of concentrations, if no nucleic acid was present. The emissions were acquired at various time points after commencement of incubation, ranging from 5 minutes to 24 hours using the Genexus® Analyzer. Supplemental Figure S1 showed the 5 minute, 65 minute and 24 hour emissions as a function of YOYO-1 concentration.

At concentrations between 50 and 350 nM, YOYO-1 facilitated probe:probe structures which formed by the 5 minute time point and were stable thereafter for at least 24 hours, as indicated by near uniform fluorescent emissions. At concentrations between 350 and 900 nM, YOYO-1 facilitated and signalled probe:probe structures which appeared to dissipate over time. At concentrations of 900 nM YOYO-1 or more, probe:probe complexes appeared not to occur, or fluorescent emission from such complexes was abolished, should they have formed. After 24 hours of incubation, concentrations of 700 nM YOYO-1 or more appeared to abolish probe:probe complexes, or emissions from them, should they have remained. We speak in absolute terms for convenience and to express the general meaning of our data. We do not suggest that better instrumentation or more precise experiments will not generate data, which will, at the margin, qualify our statements.

The Supplemental Figure S1 data will be very disturbing, if one's only experience with intercalators has been to use a fixed amount of intercalator to quantitate an unknown amount of duplex DNA. In addition to the non-linear, fluorescence pattern alluded to above, it is as well surprising that a lesser quantity of YOYO-1 might fluoresce more brightly than a greater quantity in the presence of a constant amount of DNA. Nevertheless, it was an observable, highly reproducible and hence incontrovertible result.

The maximum fluorescent emission in the presence of 3.2 pmoles of probe oligos arose when 100 nM of YOYO-1 was present. Molecules of YOYO-1 intercalated with duplex genomic DNA, as shown below, did not emit very intensely per molecule, as compared to those intercalated in probe:probe complexes. The inference may be fairly drawn that slight amounts of YOYO-1 complexed with homologously paired probes in a triplex assay test mixture will produce unwanted background emissions which may be significantly more intense than emissions from like amounts of YOYO-1 intercalated with duplex genomic DNA. Such probe:probe associated emissions will tend to minimize the triplex-associated fluorescence (TAF) which can only be ascertained after subtraction of the corresponding “probe plus YOYO-1” control emission value. So as to not double count YOYO-1 background emissions, we arrived at TAF by subtracting from the total emission from the reaction mixture the concurrently acquired “probe plus YOYO-1” control emission intensity, while not subtracting the “target plus YOYO-1” control emission intensity.

The data presented in Supplemental Figure S1 was obtained from a reaction mixture containing 40 mM TMA-Cl, a kosmotropic cation. Supplemental Figure S2 compared YOYO-1 emissions acquired after 5 minutes of incubation in the presence or absence of 40 mM TMA-Cl, when constant amounts of the DNA oligo probes were present and concentrations of YOYO-1 varied. TMA-Cl is a water structuring agent. It is understood to co-ordinate and bind multiple water molecules. Supplemental Figure S2 disclosed some other surprising results.

The presence of TMA-Cl in the concentration employed, facilitated a smooth and largely uninterrupted decline in YOYO-1 emissions from reaction mixtures containing increasing YOYO-1 concentrations, greater than 100 nM. Absent TMA-Cl, the reaction mixtures exhibited a constant low level of YOYO-1 emission at YOYO-1 concentrations of 400 nM or more, suggesting the absence of probe:probe complexes in the presence of those concentrations of YOYO-1 or the inability of those concentrations of intercalated YOYO-1s to emit light upon irradiation. It is surprising that a low concentration of TMA-Cl could have such a striking effect on YOYO-1 emissions and/or probe:probe complex formation in the presence of an overwhelming greater number of water molecules. From this data it appeared that, absent TMA-Cl, YOYO-1, at concentrations above 400 nM, disrupted the ability of probes to bind one another homologously to create an anhydrous environment hospitable to YOYO-1 intercalation and hence YOYO-1 emissions.

Supplemental Figure S3 displayed 5 minute incubation data from reaction mixtures, with and without 40 mM TMA-Cl, where 3.2 pmoles of dsDNA bglIR probes were incubated with YOYO-1 concentrations from 50 nM to 1000 nM, at 50 nM intervals. The dsDNA probes were comprised of antisense probe 1 and a complementary 25-mer antiparallel sense strand to which it was annealed in the presence of 100 mM NaCl. Here the objective was to plot the emissions from varying YOYO-1 concentrations present with a defined amount of identical short duplex DNA. The data for the 65 minute incubation time point in each experiment were virtually identical and the 24 hour time point data were similar in each experiment. In Supplemental Figure S3 we saw, as we did with the experiments with ssDNA oligo probes, that maximal emission levels occurred, surprisingly, with low YOYO-1 concentrations present and hence low levels of YOYO-1 intercalation. At increasing YOYO-1 concentrations above 200 nM, TMA-Cl facilitated a steady, essentially linear, decline in emission intensities. This decline was unexpected. We did not conclude that the short duplexes were saturated in the presence of 200 nM YOYO-1. In the reaction mixtures without TMA-Cl, increasing YOYO-1 concentrations from 50 to 150 nM YOYO-1 resulted in increased emissions but there was an abrupt emission decline when concentrations between 200 and 300 nM YOYO-1 were present, and in the presence of yet greater YOYO-1 concentrations, declining emissions were produced.

Supplemental Figure S4 disclosed data from experiments where reaction mixtures, with and without 40 mM TMA-Cl, contained 2 ng purified human genomic duplex DNA (gDNA). Concentrations of YOYO-1 varying from 50 to 1000 nM, at 50 nM intervals were added. The Supplemental Figure S4 data was obtained after 5 minutes of incubation. The 5 minute emission intensities from reaction mixtures containing various concentrations of YOYO-1 were virtually identical at the 24 hour time point (data not shown). In the presence of TMA-Cl, the data generally indicated emissions, whose intensities increased, more or less linearly, by about 50% over the tested range of increasing YOYO-1 concentrations. In the absence of TMA-Cl, the emissions from a constant amount of human gDNA in the presence of increasing YOYO-1 concentrations above 200 nM, tended to decrease at a generally linear way. This data too would not be expected by anyone conversant with using a constant amount of intercalating dye to quantitate an unknown amount of duplex DNA. It showed how TMA-Cl, at slight concentration, may affect emission from duplex intercalated YOYO-1s. All of the foregoing data provides much to be pondered. Almost nothing related to YOYO-1 emissions from low concentrations appears to occur as one would expect. Fortunately, in spite of all this, YOYO-1 makes possible a homogeneous triplex SNP assay of duplex genomic DNA sample which is directly detectable.

Supplemental Figure S5 disclosed data from reaction mixtures in which perfect or 1 bp mismatch triplexes were formed using our standard 3.2 pmole/80 µl concentration of 25-mer oligo probes and 2 ng of wild-type human genomic dsDNA, 40 mM TMA-Cl and 600 nM YOYO-1. To identify triplex-associated emissions, fluorescence emission values from the reaction mixtures were corrected for background fluorescence by subtracting the “probe plus YOYO-1” control emission intensities acquired at the incubation time points at which reaction mixture emissions were also acquired. The emission values giving rise to Supplemental Figure S5 data are produced in Supplemental Table S11. The data disclosed that the triplex-associated emissions of the mismatched triplex, under the conditions of the reaction, were less than that of the matched triplex. Supplemental Figure S6 presented the triplex-associated emissions from triplex assay mixtures when 500 nM YOYO-1 and 45 mM TMA-Cl were present. The emission values giving rise to Supplemental Figure S6 are produced in Supplemental Table S12. Supplemental Figure S6 disclosed very significant TAF single base pair assay discrimination, far in excess of that produced when 600 nM YOYO-1 and 40 mM TMA-Cl concentrations were used. This data disclosed that YOYO-1 concentration must be matched with other reagents present in the reaction mixture to carry out optimally sensitive and optimally signalled triplex SNP assays of genomic samples employing YOYO-1 as the promoter and reporter of the assay. All such matching must be explored empirically.

The foregoing provides data disclosing some of the puzzling but adventitious aspects of YOYO-1 emissions. Complexed YOYO-1 emissions can be remarkably intense at very dilute concentration which may contribute high non-specific background emissions. YOYO-1 emission variability produced by the class of nucleic acid complex into which it is intercalated makes possible a very sensitive and homogeneous assay for the presence of low copy pathogens or SNPs in human genomic samples. It is of great importance to realize that the YOYO-1 emission per molecule is immensely variable depending on the nucleic acid structure into which it is intercalated, and depends as well upon relative and local YOYO-1 and reagent concentrations. It is readily apparent that the YOYO-1 promoted and signalled triplex assay is a phenomenon strongly related to hydration because kosmotropic agents, such as TMA-Cl, can contribute very significantly to the TAF. YOYO-1 causes whatever nucleic acids are present and capable of providing multi-stranded anhydrous environments for YOYO-1 intercalation, to set up in 5 minutes or less at room temperature and thereafter to remain remarkably stable.

### Discussion

Molecular diagnostics of nucleic acids currently rely almost exclusively on a PCR amplification step. Due to the complexity of PCR optimization, the risk of contaminant amplification, the presence of known or unknown PCR inhibitors in reaction mixtures, and the inherent replication error rate, the potential for misdiagnosis when using PCR based diagnostic methodologies is significant [15], [16]. Overcoming or coping with all of these factors adds to the cost of using the PCR method.

The well known sequence requirements of “canonical triplex” formation have been heretofore understood to preclude development of molecular diagnostic methods whereby base pairs in stable heteropolymeric duplex samples may be assayed with base specific resolution using third strand probes.

We have developed a Genomic Assay® providing direct detection of heteropolymeric triplex formation between native genomic dsDNA and ssDNA probes, which eliminates reliance on PCR amplification of genomic samples. The Genomic Assay® results presented here clearly demonstrate the capability to assay human genomic dsDNA homogeneously, in solution, at RT, after as little as 5 minutes of incubation, using unlabeled ssDNA probes and YOYO-1. Such heteropolymeric triplexes can be formed to detect sequences, SNPs, deletions, insertions and repeated sequences in genomic samples, or to detect the presence or genotype of another organism or pathogen in a sample also containing human genomic dsDNA. The assay provides for the direct detection of a signal related to the base specific triplex complex formed by the duplex target sequence and probe in a homogeneous solution.

The Genomic Assay® reported herein depends upon YOYO-1 bis-intercalation rendering the duplex target into a locally de-condensed conformation [23], which allows for specific base recognition and detectable binding to occur between a heteropolymeric base sequence of a strand in the duplex and the heteropolymeric base sequence of the probe strand. The Ingeneus Triplex® and the Genomic Assay® are therefore conformation dependent, whereas the “canonical triplex” is homopurine or homopyrimidine sequence dependent. The base specific heteropolymeric triplex assay we report is therefore *sui generis*. The base specific heteropolymeric complex formed in the assay reported here approximates the triple strand intermediates which are postulated by various models of homologous recombination. Homologous recombination *in vivo* requires the action of recombination proteins, such as RecA, to locally de-condense the duplex so as to facilitate specific heteropolymeric recognition and binding of the mixed base third strand. RecA's effect on binding duplex is to locally de-condense the duplex 1.5 fold [24], [25]. YOYO-1, which is a non-specific bis-intercalator as used by us, is known to locally de-condense the duplex 1.3 fold [23] upon intercalation. We accordingly propose that YOYO-1 causes duplex DNA to be rendered into a conformation suitable for the specific recognition and binding of a heteropolymeric probe strand to a sequence of bases present in the duplex target.

There are two models of homologous recombination which seem relevant to the understanding of the Genomic Assay® and the base specific Ingeneus Triplex® upon which it depends. The model first proposed involves third strand recognition of duplex base sequence in the major groove of the duplex [26]. That model has been elaborated since [27]–[29]. The other model involves third strand recognition of duplex base sequence in the minor grove of the duplex [30]. Our references regarding each of the two homologous recombination models referred to are representative only and are not exhaustive of the groups working on such models and their published results.

In most cases, the triplex-associated fluorescent emission signal we observe could be detected within the first 5 min of incubation (Figures 1–5). While triplex-associated fluorescent emissions from homogeneous reaction mixtures containing perfectly matched genomic triplexes were relatively high, the triplex-associated fluorescent emissions from reaction mixtures comprising mismatched triplexes were lower and could be very low (Figures 2–5 and Tables 2–4). The triplex-associated fluorescent emissions observed from SNP assay mismatched triplexes were often between 80% to 100% less than those observed from the respective perfectly matched triplexes. The observation that the emissions attributable to triplex complexes formed with hgDNA samples after 5 min of incubation, and were stably detected throughout extended incubations, strongly suggests that equilibrium of triplex formation had been substantially achieved after only five min of incubation at RT. Time constraints imposed by manual handling steps have precluded our monitoring of emissions attributable to triplex formation after incubations shorter than 5 min.

Reaction mixtures and protocols can be selected in which changes in fluorescent emission levels between perfect match triplexes and mismatch triplexes can allow for better discrimination based upon “waxing and waning” of emitted signals monitored over time. The waxing and waning emission pattern may be enhanced by the addition of selected concentrations of one or more kosmotropic cations, added separately or in combination, to an assay reaction mixture (Figure 5 and data not shown). Additionally, there are advantages in carrying out assays in duplicate employing varied protocols or reagents, so as to obtain signals whose respective characteristics confirm the scoring of the sample. When assaying genomic targets, it is very useful to have fluorescence based methods, which are not dependent merely on relative strength of emission intensities, but which also encompass rates and direction of change of emissions over time.

The use of mathematical models to understand the thermodynamics and kinetics of matched and mismatched duplex hybridization, as time and concentration dependent, has recently been reported [31]. Transitory phases in which mismatched duplexes are initially more numerous than matched duplexes are theorized with mismatched duplexes disassociating as the reaction mixture comes to equilibrium. Our waxing and waning methods are congruent with such observations and theorizing.

The Genomic Assay® can be very efficient and specific, regardless whether YOYO-1 or genomic dsDNA has been added last to the reaction mixtures (Figures 5A and 5B). This will allow the Genomic Assay® to be carried out using kits containing all reagents specific for the assay to be conducted, to which the test genomic dsDNA is added last.

It is remarkable how easily 25-mer heteropolymeric specific triplexes form in the presence of YOYO-1 and that they may be directly and homogeneously detected in a reaction mixture containing human genomic dsDNA. The number of YOYO-1 molecules present in a typical Genomic Assay® reaction mixture as presented in this report is limiting and the number of potential binding sites in a human genomic target is in excess, as is the potential of ssDNA oligonucleotide probes to form parallel homologous complexes stabilized and signaled by intercalated YOYO-1s (Daksis and Erikson, unpublished observations). Upon introduction of human genomic dsDNA to a reaction mixture containing YOYO-1 and ssDNA oligonucleotide probes, it appears that YOYO-1 intercalation into duplex DNA and matched triplexes is favoured over YOYO-1 interaction with probe:probe complexes. It is possible that the greatly increased fluorescence emitted by perfectly matched triplex structures, detectable above the YOYO-1 emission from genomic dsDNA background, may be a result of mutual donor:donor energy migration between stimulated YOYO-1 molecules incorporated in the enhanced anhydrous conditions of the triplex structure.

The Genomic Assay® may also be performed with labels other than YOYO-1 used free in solution, or in formats other than in bulk solution (Daksis and Erikson, unpublished observations).

In conclusion, we have developed a rapid, homogeneous assay based on detectable heteropolymeric triplex formation between native genomic dsDNA and ssDNA probes, which can eliminate recourse to PCR amplification based methods. The Genomic Assay® allows the direct detection of low copy pathogen genomes present in a sample also containing human genomic duplex DNA, or the direct detection of SNPs in human genomic duplex DNA. Our assay constitutes a hybridization assay which is fundamentally different than all previously developed hybridization assays, since no denaturation of the duplex DNA is required prior to binding of the ssDNA probe. Furthermore, the presence of long stretches of homopurine or homopyrimidine sequences in the binding region of the duplex DNA, as necessary for “canonical triplex” formation, is not required in our assay. Provided a suitable local de-condensation of the duplex target is achieved, specific heteropolymeric triplexes readily form with third strand probes with base pair resolution. The Genomic Assay® herein presented depends upon YOYO-1, a bis-intercalator, to both de-condense the duplex and to signal the presence of the specific triplex of the assay. The heteropolymeric triplexes promoted by YOYO-1 approximate the heteropolymeric homologous recombination triplex intermediates, which occur *in vivo* under the action of recombination proteins, such as RecA. Homologous recombination models propose that mixed base ssDNA sequences bind either in the major or minor groove of the duplex. It is accordingly no longer necessary to denature genomic duplex DNA to be able to assay it for the presence of a sequence of interest or a SNP, nor is it necessary to perform a separation step if one wishes to employ optical signals to assay a highly complex target in bulk solution.

## Materials and Methods

### Single-stranded oligonucleotide probes

Sequences of the ssDNA probes used are listed in Table 1. All oligonucleotide probes were synthesized on a DNA synthesizer, cartridge purified and dissolved in ddH_2_O at a concentration of 1 pmole/µl. Probe 1 was complementary to a segment of the sense strand of the Bgl I restriction endonuclease (*bglIR*) gene in *Bacillus globigii* [32]. Probe 2 was complementary to a segment of the antisense strand of the *erg* gene in *Drosophila melanogaster* [17]. Probes 3 to 30 were derived from various exons and/or introns of the human cystic fibrosis transmembrane conductance regulator gene (*CFTR*) [33]. Probes 31 and 32 were derived from exon 4 of the human methylenetetrahydrofolate reductase (*MTHFR*) gene [22]. Probes 33 and 34 were derived from exon 10 of the human factor V gene [20].

### Genomic sample isolation

*Bacillus globigii* (BG) genomic dsDNA was isolated from lysed vegetative BG cells using a QIAamp DNA blood mini-purification kit as per the manufacturer's instructions (Qiagen, Mississauga, Canada). *Drosophila* genomic dsDNA was purified from *Drosophila* wild-type Canton-S and mutant *sei^ts1^* strains by multiple phenol-chloroform extractions and kindly provided by Dr. Barry Ganetzky (University of Wisconsin, Madison, WI, USA). Human genomic dsDNA was extracted from human blood using a QIAamp DNA blood purification kit (Qiagen, Mississauga, Canada) or from human saliva using an Oragene DNA collection kit (DNA Genotek, Ottawa, Canada) as per the manufacturers' instructions. The concentration of each genomic dsDNA sample was determined by UV spectroscopy.

### Genomic Assay®

Different concentrations of genomic dsDNA were assayed, dependent on the source of genomic DNA. Unless otherwise indicated, the triplex binding reaction mixtures (80 µl) contained the following: between 4 ng and 75 pg of human genomic dsDNA, 3.2 pmoles of 25-mer ssDNA probe, 22.5 mM Tris-borate, pH 8.3, 0.5 mM EDTA (0.5×TBE) and 500 nM YOYO-1. YOYO-1, a hydrophobic bis-intercalator quenched by aqueous medium, was supplied by Molecular Probes (Eugene, Oregon, USA). Following a 5 min incubation at room temperature (RT) (22°C), the reaction mixtures were placed into separate wells of a Corning No Bind Surface 384-well plate (black with clear bottom, with universal lid) and irradiated through the bottom of the multi-well plate by the Genexus® Analyzer. The Genexus® Analyzer employs a Melles Griot 15 mW argon ion laser, Model 35-IMA-415-120, configured to deliver about 84 W/cm^2^/sec radiation at a wavelength of 488 nm to each sample. The Genexus® Analyzer, manufactured by Biomedical Photometrics Inc. (Waterloo, Ontario, Canada), utilizes Macroview software, which controls the scanning parameters of the confocal laser and provides quantitative analysis of scanned samples in the multi-well plates. Irradiation occurred at a sampling interval of 60 microns at settings of 20 hertz, 30–34% PMT and 10 µA/V sensitivity. At these scanning parameters, approximately 44,000 pixel emissions were measured per assay and averaged. Fluorescent emissions were also monitored over time. The Genexus® Analyzer allows 384 test samples to be scanned in approximately 2 min. Care must be taken in selecting means of stimulating YOYO-1 emission. YOYO-1 acts as a photocleavage agent if stimulated with overly energetic radiation. We have empirically determined that light-emitted diode (LED) irradiation, or laser irradiation of 84 W/cm^2^/sec can be optimal. Instruments powered by “bright light” sources, such as Xenon bulbs, that generate pulsed or continuous bright light, which is subsequently filtered, have not proved useful to carry out the Triplex Assay® using YOYO-1.

## Supporting Information

Figure S1. Fluorescence emissions from varied YOYO-1 concentrations and a 25-mer ssDNA probe. 3.2 pmoles of a 25-mer ssDNA probe was reacted with various concentrations of YOYO-1, ranging from 50 nM to 1000 nM, at 50 nM intervals, in the presence of 0.5×TBE and 40 mM TMA-Cl. Fluorescent emissions of the reaction mixtures (80 ul) were monitored with the Genexus Analyzer at a setting of 32% PMT after 5, 15, 25, 35, 45, 55 and 65 minutes, and 24 hours of incubation at RT. Intensity of fluorescence is plotted as a function of YOYO-1 concentration for the probe:probe complexes formed after 5 min of incubation (1), 65 min of incubation (2) or 24 hours of incubation (3).
(0.01 MB PDF)

Figure S2. Comparison of the fluorescence emissions from varied YOYO-1 concentrations and a 25-mer ssDNA probe in the presence or absence of a kosmotropic agent. 3.2 pmoles of a 25-mer ssDNA probe was reacted with various concentrations of YOYO-1, ranging from 50 nM to 1000 nM, at 50 nM intervals, in the presence of 0.5×TBE, and in the presence or absence of 40 mM TMA-Cl. Fluorescent emissions of the reaction mixtures (80 ul) were monitored with the Genexus Analyzer at a setting of 32% PMT after 5, 15, 25, 35, 45, 55 and 65 minutes, and 24 hours of incubation at RT. Intensity of fluorescence is plotted as a function of YOYO-1 concentration for the probe:probe complexes formed after 5 min of incubation in the presence (1) or absence (2) of 40 mM TMA-Cl.
(0.01 MB PDF)

Figure S3. Comparison of the fluorescence emissions from varied YOYO-1 concentrations and a 25-mer dsDNA in the presence or absence of a kosmotropic agent. 3.2 pmoles of a 25-mer dsDNA was reacted with various concentrations of YOYO-1, ranging from 50 nM to 1000 nM, at 50 nM intervals, in the presence of 0.5×TBE, and in the presence or absence of 40 mM TMA-Cl. Fluorescent emissions of the reaction mixtures (80 ul) were monitored with the Genexus Analyzer at a setting of 32% PMT after 5, 15, 25, 35, 45, 55 and 65 minutes, and 24 hours of incubation at RT. Intensity of fluorescence is plotted as a function of YOYO-1 concentration for the dsDNA:YOYO-1 complexes formed after 5 min of incubation in the presence (1) or absence (2) of 40 mM TMA-Cl.
(0.01 MB PDF)

Figure S4. Comparison of the fluorescence emissions from varied YOYO-1 concentrations and human genomic dsDNA in the presence or absence of a kosmotropic agent. Human genomic dsDNA was extracted from blood as described in the text. Two ng of wild-type human genomic dsDNA (approximately 302 copies) was reacted with various concentrations of YOYO-1, ranging from 50 nM to 1000 nM, at 50 nM intervals, in the presence of 0.5×TBE, and in the presence or absence of 40 mM TMA-Cl. Fluorescent emissions of the reaction mixtures (80 ul) were monitored with the Genexus Analyzer at a setting of 32% PMT after 5, 15, 25, 35, 45, 55 and 65 minutes, and 24 hours of incubation at RT. Intensity of fluorescence is plotted as a function of YOYO-1 concentration for the dsDNA:YOYO-1 complexes formed after 5 min of incubation in the presence (1) or absence (2) of 40 mM TMA-Cl.
(0.01 MB PDF)

Figure S5. Assays of human genomic dsDNA for CFTR 3849+10kbC→T (1 bp A–C mismatch) in the presence of 600 nM YOYO-1 and 40 mM TMA-Cl. Human genomic dsDNA was extracted from blood as described in the text. Two ng of wild-type human genomic dsDNA (approximately 302 copies) was reacted at RT with 3.2 pmoles of either wild-type or mutant ssDNA probe in the presence of 0.5×TBE, 40 mM TMA-Cl and 600 nM YOYO-1. YOYO-1 was added last to the reaction mixtures. Reaction mixtures (80 ul) were irradiated as described in the text and analyzed for fluorescent emission. The intensity of triplex-associated fluorescence is plotted as a function of incubation time for each sample analyzed. The samples consist of perfectly matched triplex (1) and mismatched triplex (2) as indicated for CFTR 3849+10kbC→T.
(0.01 MB PDF)

Figure S6. Assays of human genomic dsDNA for CFTR 3849+10kbC→T (1 bp A–C mismatch) in the presence of 500 nM YOYO-1 and 45 mM TMA-Cl. Human genomic dsDNA was extracted from blood as described in the text. Two ng of wild-type human genomic dsDNA (approximately 302 copies) was reacted at RT with 3.2 pmoles of either wild-type or mutant ssDNA probe in the presence of 0.5×TBE, 45 mM TMA-Cl and 500 nM YOYO-1. YOYO-1 was added last to the reaction mixtures. Reaction mixtures (80 ul) were irradiated as described in the text and analyzed for fluorescent emission. The intensity of triplex-associated fluorescence is plotted as a function of incubation time for each sample analyzed. The samples consist of perfectly matched triplex (1) and mismatched triplex (2) as indicated for CFTR 3849+10kbC→T.
(0.01 MB PDF)

Table S1. Assays of Bacillus globigii genomic dsDNA in the presence or absence of 2 ng human genomic dsDNA. 100 copies of Bacillus globigii genomic dsDNA are detected by triplex assay in reaction mixtures containing 300 nM YOYO-1 in the presence or absence of 2 ng human genomic dsDNA.
(0.05 MB DOC)

Table S2. Comparison of triplex assays of human genomic dsDNA using 15-mer, 20-mer or 25-mer ssDNA probes. Triplex assays of human genomic dsDNA for CFTR delta F508 using 15-mer, 20-mer or 25-mer ssDNA probes demonstrate optimal probe length under the conditions employed, to be 25 bases.
(0.06 MB DOC)

Table S3. Comparison of triplex assays of human genomic dsDNA using 20-mer, 25-mer or 30-mer ssDNA probes. Triplex assays of human genomic dsDNA for MTHFR C677T using 20-mer, 25-mer or 30-mer ssDNA probes demonstrate optimal probe length under the conditions employed, to be 25 bases.
(0.06 MB DOC)

Table S4. Assays of varying concentrations of human genomic dsDNA for Factor V Leiden (1 bp G–T mismatch). The specificity of the triplex assay in detecting FVL G1691A in mismatched triplexes is demonstrated over a broad range of human genomic dsDNA concentrations.
(0.07 MB DOC)

Table S5. Assays of varying concentrations of human genomic dsDNA for CFTR 2789+5G→A (1 bp T–G mismatch). The specificity of the triplex assay in detecting CFTR 2789+5G→A in mismatched triplexes is demonstrated over a broad range of human genomic dsDNA concentrations.
(0.08 MB DOC)

Table S6. Assays of human genomic dsDNA (purified from blood) for CFTR 2789+5G→A (1 bp T–G mismatch). The specificity of the triplex assay in detecting CFTR 2789+5G→A in mismatched triplexes comprising human genomic dsDNA purified from blood is demonstrated.
(0.04 MB DOC)

Table S7. Assays of human genomic dsDNA (wild-type homozygous or mutant heterozygous samples purified from blood) for MTHFR C677T. The specificity of the triplex assay in assaying wild-type homozygous and mutant heterozygous human genomic dsDNA samples for MTHFR C677T is demonstrated.
(0.06 MB DOC)

Table S8. Assays of human genomic dsDNA (mutant homozygous sample purified from blood) for MTHFR C677T. The specificity of the triplex assay in assaying mutant homozygous human genomic dsDNA samples for MTHFR C677T is demonstrated.
(0.05 MB DOC)

Table S9. Assays of varying concentrations of human genomic dsDNA for MTHFR C677T (1 bp C–A mismatch). The specificity of the triplex assay in detecting MTHFR C677T in mismatched triplexes is demonstrated over a broad range of human genomic dsDNA concentrations.
(0.07 MB DOC)

Table S10. Assays of human genomic dsDNA employing varying protocols. The specificity of the triplex assay in detecting CFTR 3849+10kbC→T in mismatched triplexes is demonstrated in reaction mixtures containing a kosmotropic agent. Two reaction protocols are compared.
(0.05 MB DOC)

Table S11. Assays of human genomic dsDNA for CFTR 3849+10kbC→T (1 bp A–C mismatch) in the presence of 600 nM YOYO-1 and 40 mM TMA-Cl. The emission values giving rise to Supplementary Figure S5 data are shown. The specificity of the triplex assay in detecting CFTR 3849+10kbC→T in mismatched triplexes is demonstrated in reaction mixtures containing 600 nM YOYO-1 and 40 mM TMA-Cl.
(0.05 MB DOC)

Table S12. Assays of human genomic dsDNA for CFTR 3849+10kbC→T (1 bp A–C mismatch) in the presence of 500 nM YOYO-1 and 45 mM TMA-Cl. The emission values giving rise to Supplementary Figure S6 data are shown. The specificity of the triplex assay in detecting CFTR 3849+10kbC→T in mismatched triplexes is demonstrated in reaction mixtures containing 500 nM YOYO-1 and 45 mM TMA-Cl.
(0.05 MB DOC)
