# Supplementary figures and images for: Heteropolymeric Triplex-Based Genomic Assay® to Detect Pathogens or Single-Nucleotide Polymorphisms in Human Genomic Samples
Source: PLoS One. 2007 Mar 21;2(3):e305. doi: 10.1371/journal.pone.0000305 (PMC1810429; doi:10.1371/journal.pone.0000305)

**FIGURE S1**

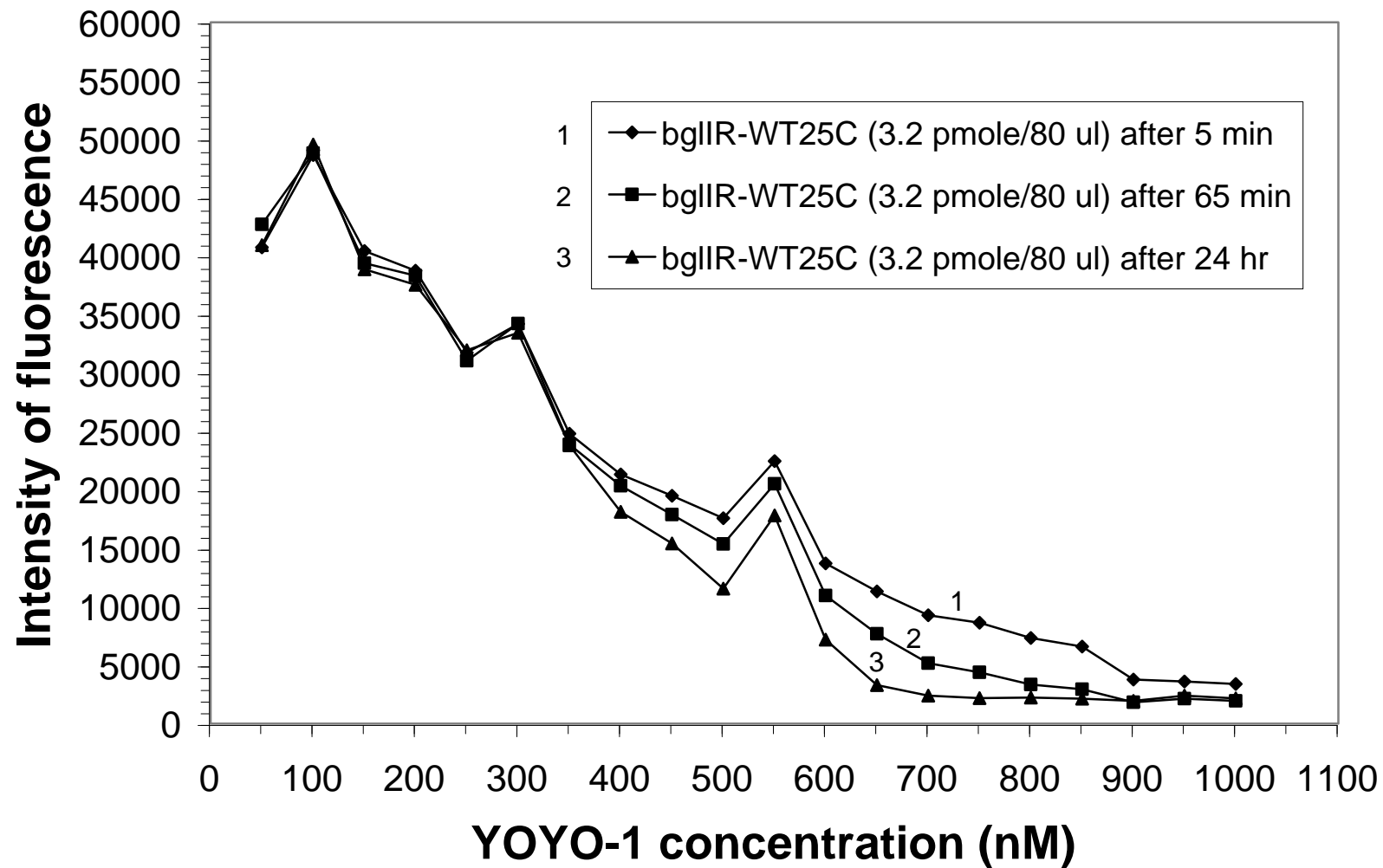

Supplement: Figure S1. — Fluorescence emissions from varied YOYO-1 concentrations and a 25-mer ssDNA probe. 3.2 pmoles of a 25-mer ssDNA probe was reacted with various concentrations of YOYO-1, ranging from 50 nM to 1000 nM, at 50 nM intervals, in the presence of 0.5×TBE and 40 mM TMA-Cl. Fluorescent emissions of the reaction mixtures (80 ul) were monitored with the Genexus Analyzer at a setting of 32% PMT after 5, 15, 25, 35, 45, 55 and 65 minutes, and 24 hours of incubation at RT. Intensity of fluorescence is plotted as a function of YOYO-1 concentration for the probe:probe complexes formed after 5 min of incubation (1), 65 min of incubation (2) or 24 hours of incubation (3). (0.01 MB PDF) [file pone.0000305.s001.pdf]

**FIGURE S2**

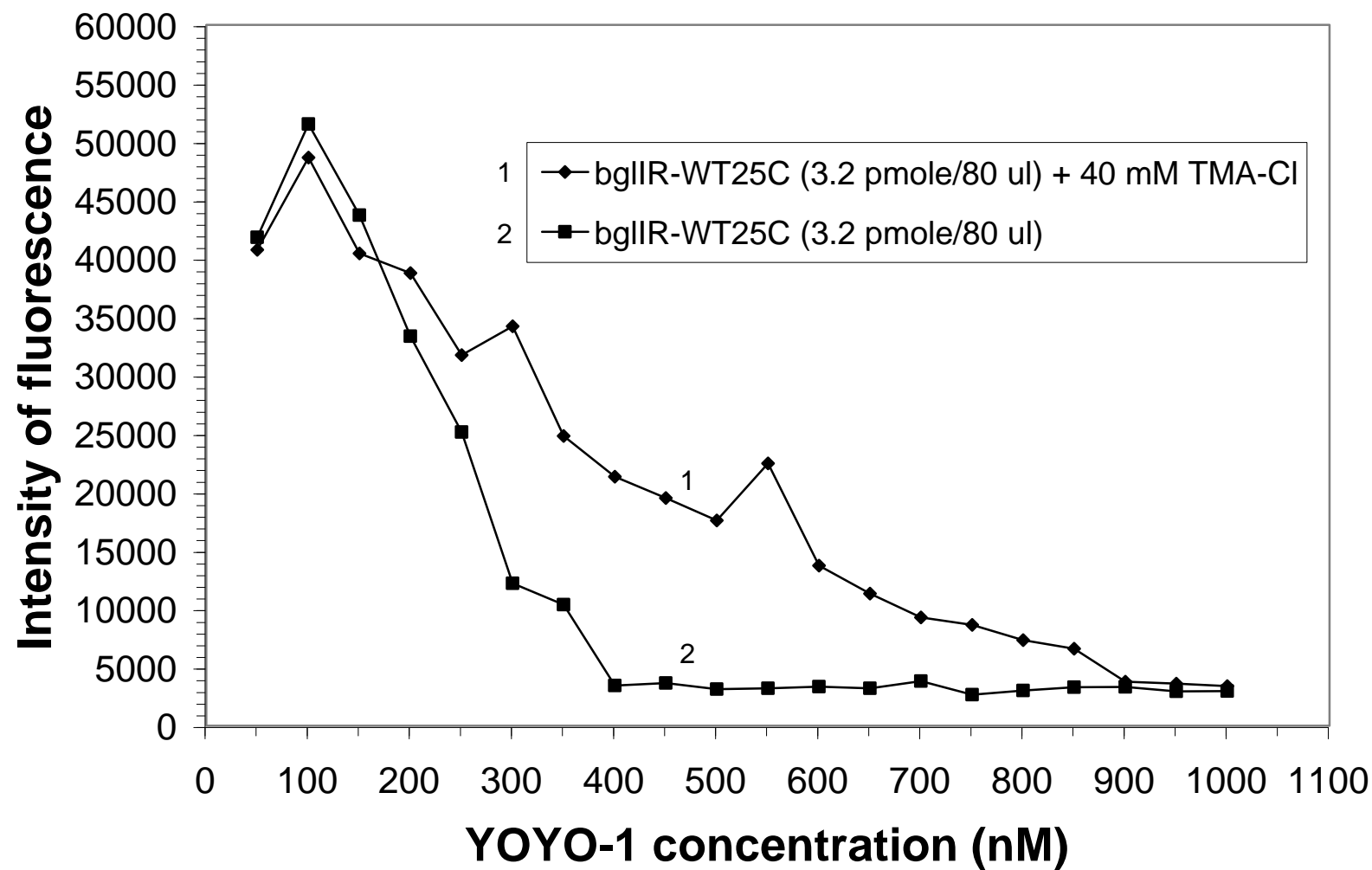

Supplement: Figure S2. — Comparison of the fluorescence emissions from varied YOYO-1 concentrations and a 25-mer ssDNA probe in the presence or absence of a kosmotropic agent. 3.2 pmoles of a 25-mer ssDNA probe was reacted with various concentrations of YOYO-1, ranging from 50 nM to 1000 nM, at 50 nM intervals, in the presence of 0.5×TBE, and in the presence or absence of 40 mM TMA-Cl. Fluorescent emissions of the reaction mixtures (80 ul) were monitored with the Genexus Analyzer at a setting of 32% PMT after 5, 15, 25, 35, 45, 55 and 65 minutes, and 24 hours of incubation at RT. Intensity of fluorescence is plotted as a function of YOYO-1 concentration for the probe:probe complexes formed after 5 min of incubation in the presence (1) or absence (2) of 40 mM TMA-Cl. (0.01 MB PDF) [file pone.0000305.s002.pdf]

**FIGURE S3**

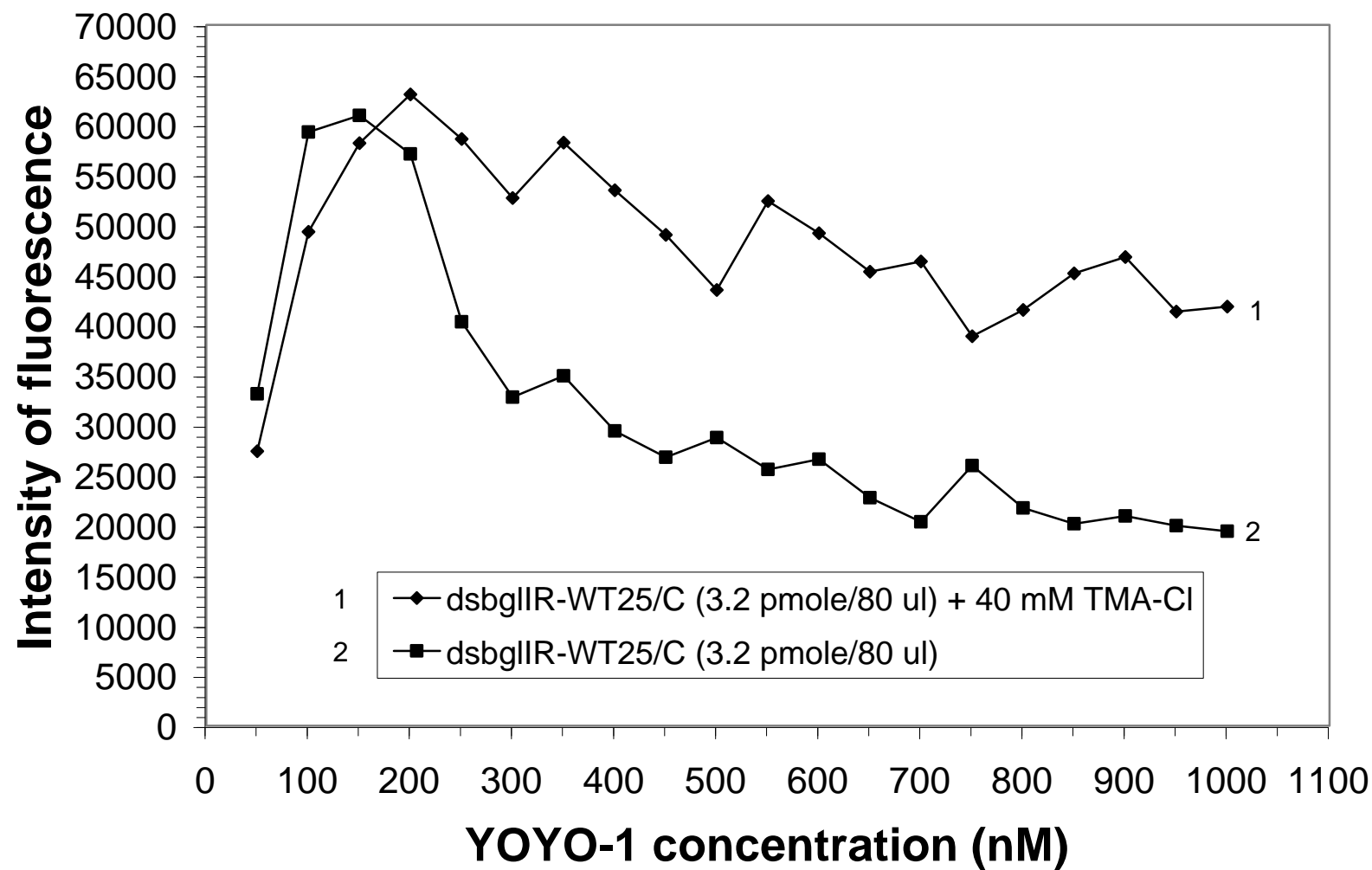

Supplement: Figure S3. — Comparison of the fluorescence emissions from varied YOYO-1 concentrations and a 25-mer dsDNA in the presence or absence of a kosmotropic agent. 3.2 pmoles of a 25-mer dsDNA was reacted with various concentrations of YOYO-1, ranging from 50 nM to 1000 nM, at 50 nM intervals, in the presence of 0.5×TBE, and in the presence or absence of 40 mM TMA-Cl. Fluorescent emissions of the reaction mixtures (80 ul) were monitored with the Genexus Analyzer at a setting of 32% PMT after 5, 15, 25, 35, 45, 55 and 65 minutes, and 24 hours of incubation at RT. Intensity of fluorescence is plotted as a function of YOYO-1 concentration for the dsDNA:YOYO-1 complexes formed after 5 min of incubation in the presence (1) or absence (2) of 40 mM TMA-Cl. (0.01 MB PDF) [file pone.0000305.s003.pdf]

**FIGURE S4**

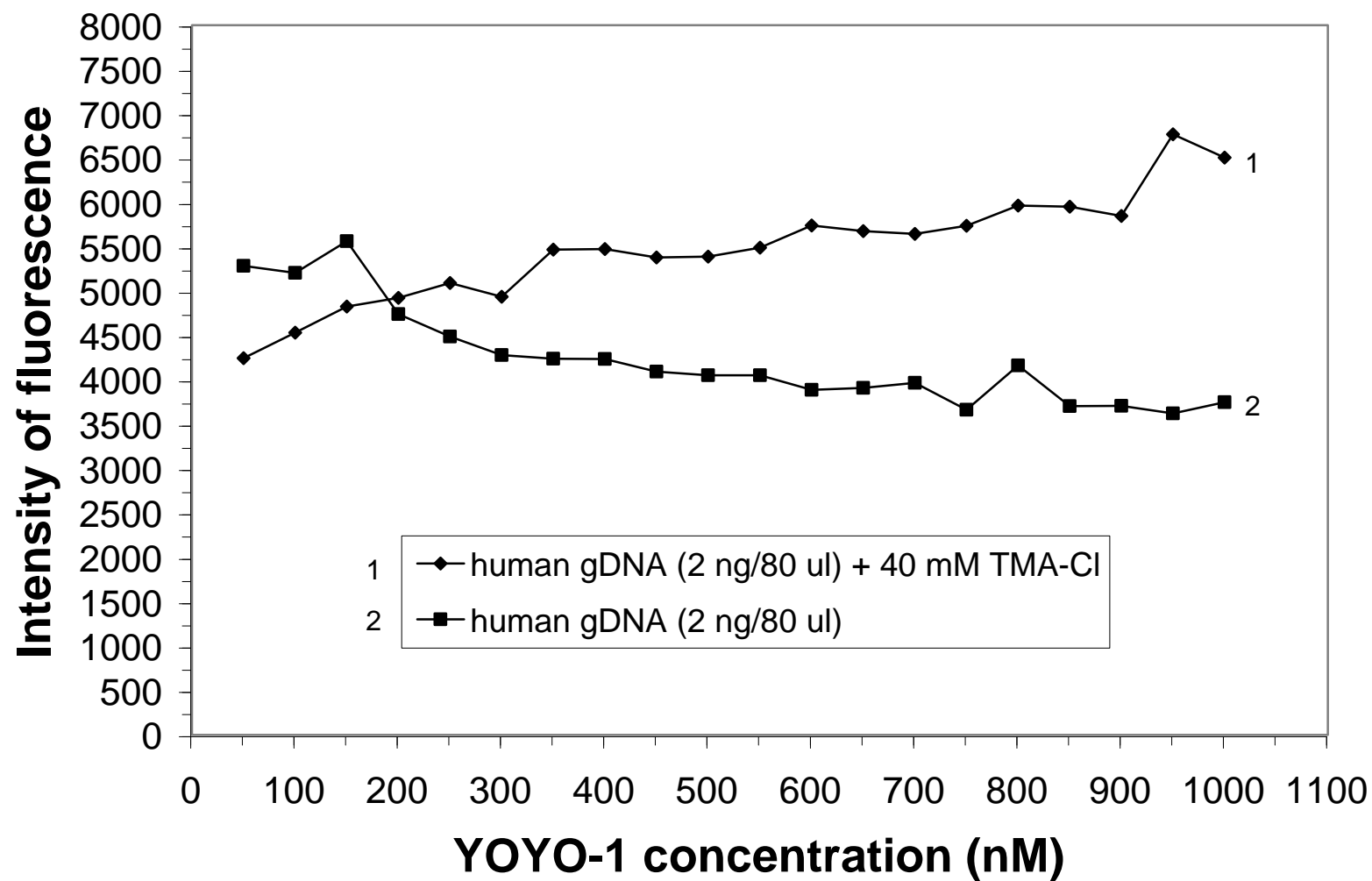

Supplement: Figure S4. — Comparison of the fluorescence emissions from varied YOYO-1 concentrations and human genomic dsDNA in the presence or absence of a kosmotropic agent. Human genomic dsDNA was extracted from blood as described in the text. Two ng of wild-type human genomic dsDNA (approximately 302 copies) was reacted with various concentrations of YOYO-1, ranging from 50 nM to 1000 nM, at 50 nM intervals, in the presence of 0.5×TBE, and in the presence or absence of 40 mM TMA-Cl. Fluorescent emissions of the reaction mixtures (80 ul) were monitored with the Genexus Analyzer at a setting of 32% PMT after 5, 15, 25, 35, 45, 55 and 65 minutes, and 24 hours of incubation at RT. Intensity of fluorescence is plotted as a function of YOYO-1 concentration for the dsDNA:YOYO-1 complexes formed after 5 min of incubation in the presence (1) or absence (2) of 40 mM TMA-Cl. (0.01 MB PDF) [file pone.0000305.s004.pdf]

**FIGURE S5**

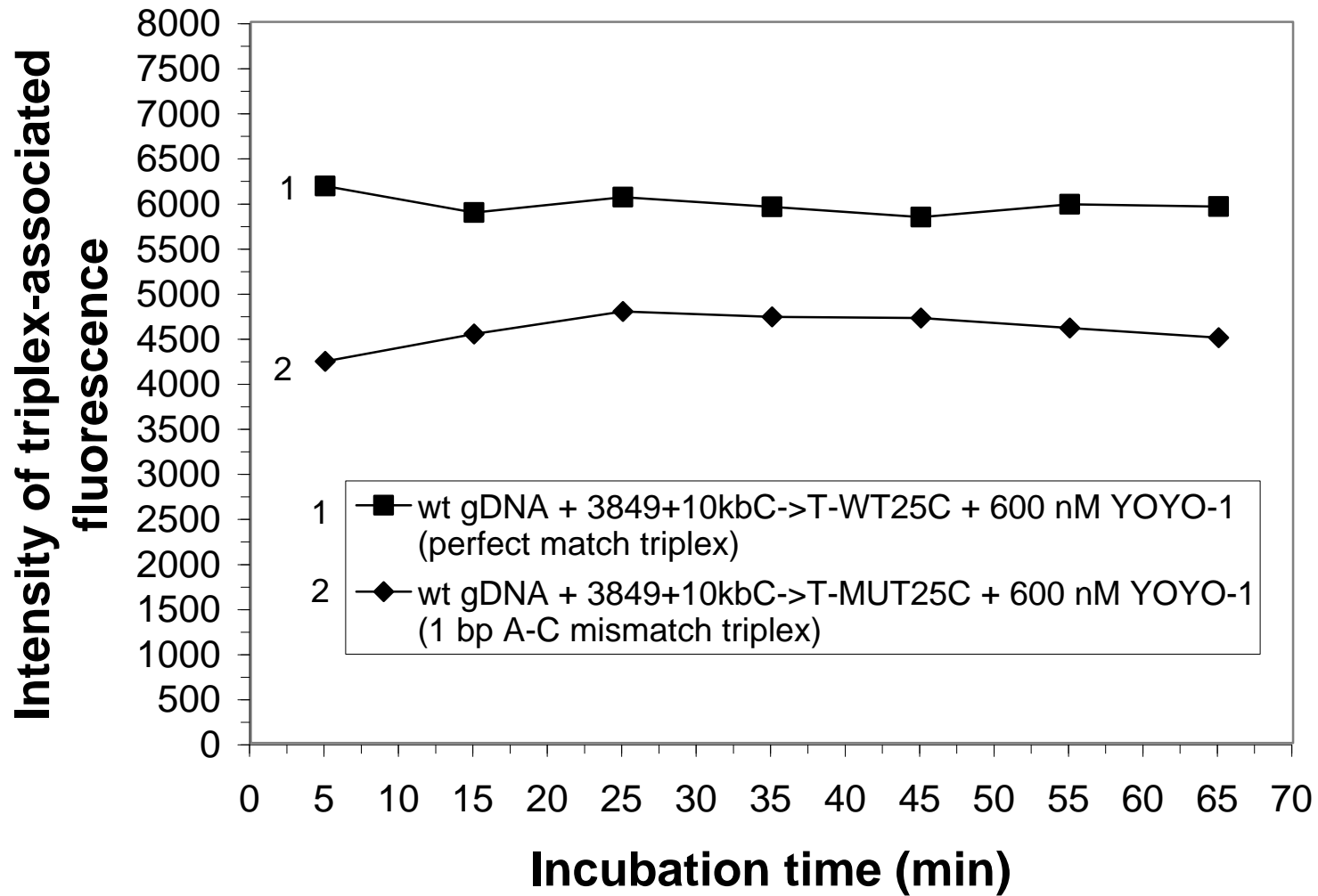

Supplement: Figure S5. — Assays of human genomic dsDNA for CFTR 3849+10kbC→T (1 bp A–C mismatch) in the presence of 600 nM YOYO-1 and 40 mM TMA-Cl. Human genomic dsDNA was extracted from blood as described in the text. Two ng of wild-type human genomic dsDNA (approximately 302 copies) was reacted at RT with 3.2 pmoles of either wild-type or mutant ssDNA probe in the presence of 0.5×TBE, 40 mM TMA-Cl and 600 nM YOYO-1. YOYO-1 was added last to the reaction mixtures. Reaction mixtures (80 ul) were irradiated as described in the text and analyzed for fluorescent emission. The intensity of triplex-associated fluorescence is plotted as a function of incubation time for each sample analyzed. The samples consist of perfectly matched triplex (1) and mismatched triplex (2) as indicated for CFTR 3849+10kbC→T. (0.01 MB PDF) [file pone.0000305.s005.pdf]

**FIGURE S6**

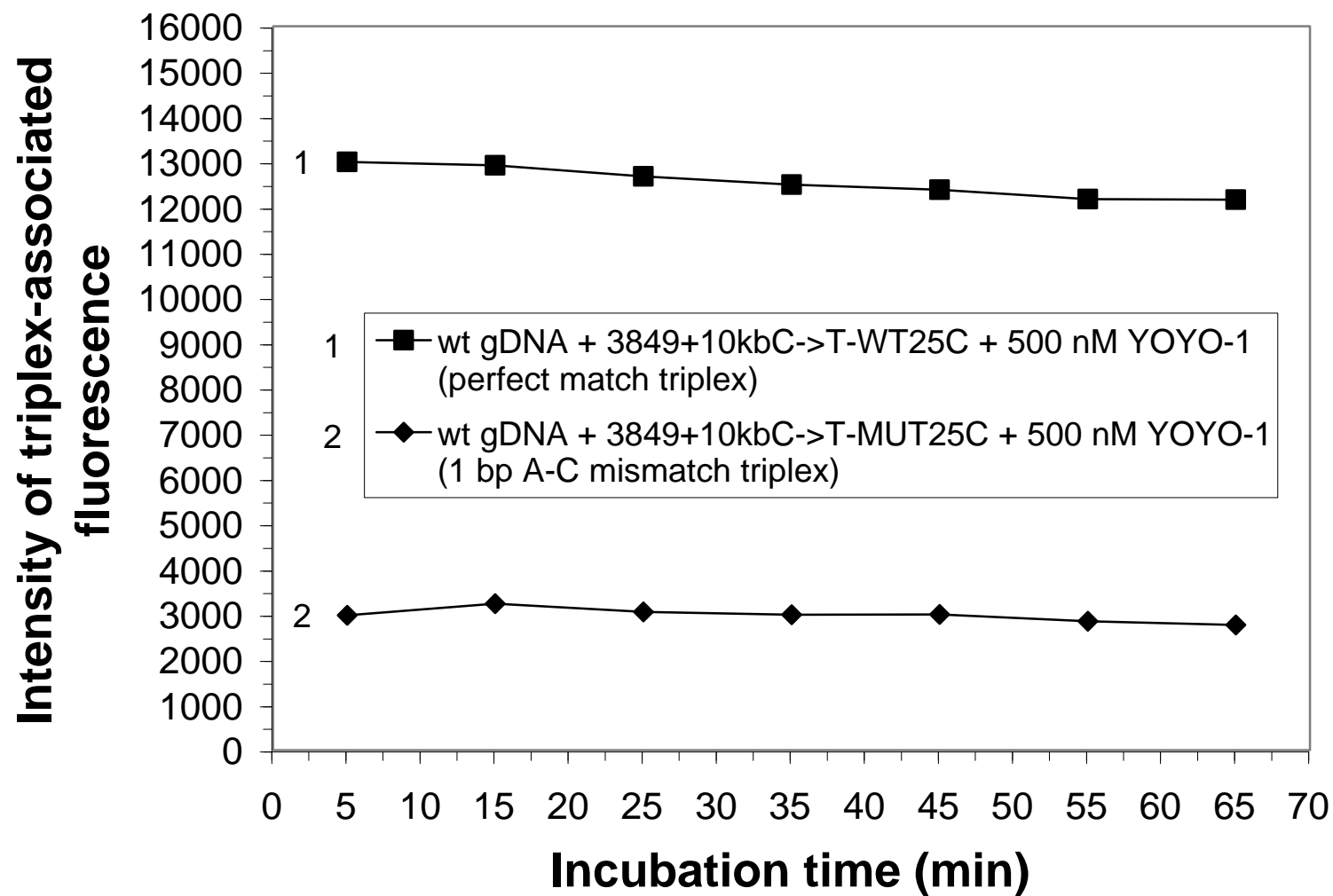

Supplement: Figure S6. — Assays of human genomic dsDNA for CFTR 3849+10kbC→T (1 bp A–C mismatch) in the presence of 500 nM YOYO-1 and 45 mM TMA-Cl. Human genomic dsDNA was extracted from blood as described in the text. Two ng of wild-type human genomic dsDNA (approximately 302 copies) was reacted at RT with 3.2 pmoles of either wild-type or mutant ssDNA probe in the presence of 0.5×TBE, 45 mM TMA-Cl and 500 nM YOYO-1. YOYO-1 was added last to the reaction mixtures. Reaction mixtures (80 ul) were irradiated as described in the text and analyzed for fluorescent emission. The intensity of triplex-associated fluorescence is plotted as a function of incubation time for each sample analyzed. The samples consist of perfectly matched triplex (1) and mismatched triplex (2) as indicated for CFTR 3849+10kbC→T. (0.01 MB PDF) [file pone.0000305.s006.pdf]
