# Supplementary material for: Heteropolymeric Triplex-Based Genomic Assay® to Detect Pathogens or Single-Nucleotide Polymorphisms in Human Genomic Samples
Source: PLoS One. 2007 Mar 21;2(3):e305. doi: 10.1371/journal.pone.0000305 (PMC1810429; doi:10.1371/journal.pone.0000305)
Supplement: Table S1. — Assays of Bacillus globigii genomic dsDNA in the presence or absence of 2 ng human genomic dsDNA. 100 copies of Bacillus globigii genomic dsDNA are detected by triplex assay in reaction mixtures containing 300 nM YOYO-1 in the presence or absence of 2 ng human genomic dsDNA. (0.05 MB DOC) [file pone.0000305.s007.doc]

**Table S1. Assays of *Bacillus globigii* genomic dsDNA in the presence or absence of 2 ng human genomic dsDNA.**

| Sample | Fluorescence on Genexus argon laser @ PMT 32 after 5 min | TAF | Fluorescence on Genexus argon laser @ PMT 32 after 15 min | TAF |
| --- | --- | --- | --- | --- |
| 1) YOYO-1 (300 nM) | 0 |  | 0 |  |
| 2) *B. globigii* gDNA (100 copies/80 ul) | 0 |  | 0 |  |
| 3) human gDNA (302 copies/80 ul) | 5321 |  | 5330 |  |
| 4) bglIR-WT25C (3.2 pmole/80 ul) (antisense) | 28663 |  | 28582 |  |
| 5) *B. globigii* gDNA (100 copies/80 ul) + bglIR-WT25C | 35536 | 6873 | 36076 | 7494 |
| 6) human gDNA (302 copies/80 ul) + bglIR-WT25C | 40732 |  | 40568 |  |
| 7)BG gDNA (100 copies) + hgDNA (302 copies) + bglIR-WT25C | 43378 | 2646 | 43237 | 2669 |

| Sample | Fluorescence on Genexus argon laser @ PMT 32 after 25 min | TAF | Fluorescence on Genexus argon laser @ PMT 32 after 35 min | TAF |
| --- | --- | --- | --- | --- |
| 1) YOYO-1 (300 nM) | 0 |  | 0 |  |
| 2) *B. globigii* gDNA (100 copies/80 ul) | 0 |  | 0 |  |
| 3) human gDNA (302 copies/80 ul) | 5225 |  | 5326 |  |
| 4) bglIR-WT25C (3.2 pmole/80 ul) (antisense) | 28103 |  | 28113 |  |
| 5) *B. globigii* gDNA (100 copies/80 ul) + bglIR-WT25C | 35810 | 7707 | 35886 | 7773 |
| 6) human gDNA (302 copies/80 ul) + bglIR-WT25C | 40238 |  | 40436 |  |
| 7)BG gDNA (100 copies) + hgDNA (302 copies) + bglIR-WT25C | 43219 | 2981 | 43390 | 2954 |

**Table S1.** Continued

| Sample | Fluorescence on Genexus argon laser @ PMT 32 after 45 min | TAF | Fluorescence on Genexus argon laser @ PMT 32 after 55 min | TAF |
| --- | --- | --- | --- | --- |
| 1) YOYO-1 (300 nM) | 0 |  | 0 |  |
| 2) *B. globigii* gDNA (100 copies/80 ul) | 0 |  | 0 |  |
| 3) human gDNA (302 copies/80 ul) | 5209 |  | 5215 |  |
| 4) bglIR-WT25C (3.2 pmole/80 ul) (antisense) | 27708 |  | 27606 |  |
| 5) *B. globigii* gDNA (100 copies/80 ul) + bglIR-WT25C | 35339 | 7631 | 35190 | 7584 |
| 6) human gDNA (302 copies/80 ul) + bglIR-WT25C | 39743 |  | 39816 |  |
| 7)BG gDNA (100 copies) + hgDNA (302 copies) + bglIR-WT25C | 42781 | 3038 | 42705 | 2889 |

| Sample | Fluorescence on Genexus argon laser @ PMT 32 after 65 min | TAF | Fluorescence on Genexus argon laser @ PMT 32 after 24 hr | TAF |
| --- | --- | --- | --- | --- |
| 1) YOYO-1 (300 nM) | 0 |  | 0 |  |
| 2) *B. globigii* gDNA (100 copies/80 ul) | 0 |  | 0 |  |
| 3) human gDNA (302 copies/80 ul) | 5119 |  | 5011 |  |
| 4) bglIR-WT25C (3.2 pmole/80 ul) (antisense) | 27517 |  | 29294 |  |
| 5) *B. globigii* gDNA (100 copies/80 ul) + bglIR-WT25C | 35074 | 7557 | 35604 | 6310 |
| 6) human gDNA (302 copies/80 ul) + bglIR-WT25C | 39480 |  | 41000 |  |
| 7)BG gDNA (100 copies) + hgDNA (302 copies) + bglIR-WT25C | 42644 | 3164 | 45031 | 4031 |

The target was *Bacillus globigii* genomic dsDNA. The 25-mer probe was bglIR-WT25C (wild-type for *B. globigii*). 300 nM YOYO-1 and 40 mM TMA-Cl were present in each sample. TAF indicates Triplex-Associated Fluorescence.
