# Supplementary material for: Heteropolymeric Triplex-Based Genomic Assay® to Detect Pathogens or Single-Nucleotide Polymorphisms in Human Genomic Samples
Source: PLoS One. 2007 Mar 21;2(3):e305. doi: 10.1371/journal.pone.0000305 (PMC1810429; doi:10.1371/journal.pone.0000305)
Supplement: Table S2. — Comparison of triplex assays of human genomic dsDNA using 15-mer, 20-mer or 25-mer ssDNA probes. Triplex assays of human genomic dsDNA for CFTR delta F508 using 15-mer, 20-mer or 25-mer ssDNA probes demonstrate optimal probe length under the conditions employed, to be 25 bases. (0.06 MB DOC) [file pone.0000305.s008.doc]

# Table S2. Comparison of triplex assays of human genomic dsDNA using 15-mer, 20-mer or 25-mer ssDNA probes.

| Sample | Fluorescence on Genexus argon laser @ PMT 34 after 5 min | TAF | % of difference relative to perfect match TAF | Fluorescence on Genexus argon laser @ PMT 34 after 15 min | TAF | % of difference relative to perfect match TAF |
| --- | --- | --- | --- | --- | --- | --- |
| 1) YOYO-1 (600 nM) | 207 |  |  | 211 |  |  |
| 2) delta F508-WT25C (3.2 pmole) (antisense) | 13680 |  |  | 9381 |  |  |
| 3) delta F508-MUT25C (3.2 pmole) (antisense) | 9169 |  |  | 8696 |  |  |
| 4) wt gDNA (500 pg) | 2723 |  |  | 2674 |  |  |
| 5) wt gDNA (500 pg) + delta F508-WT25C (perfect) | 21217 | 7537 |  | 18698 | 9317 |  |
| 6) wt gDNA (500 pg) + delta F508-MUT25C (3 bp AAG del) | 9913 | 744 | - 90.1 | 9355 | 659 | - 92.9 |
| 7) delta F508-WT20C (3.2 pmole) (antisense) | 6293 |  |  | 5826 |  |  |
| 8) delta F508-MUT20C (3.2 pmole) (antisense) | 767 |  |  | 809 |  |  |
| 9) wt gDNA (500 pg) | 2481 |  |  | 2381 |  |  |
| 10) wt gDNA (500 pg) + delta F508-WT20C (perfect) | 7155 | 862 |  | 6820 | 994 |  |
| 11) wt gDNA (500 pg) + delta F508-MUT20C (3 bp AAG del) | 1258 | 491 | - 43.0 | 1032 | 223 | - 77.6 |
| 12) delta F508-WT15C (3.2 pmole) (antisense) | 3825 |  |  | 3639 |  |  |
| 13) delta F508-MUT15C (3.2 pmole) (antisense) | 4597 |  |  | 4549 |  |  |
| 14) wt gDNA (500 pg) | 2505 |  |  | 2495 |  |  |
| 15) wt gDNA (500 pg) + delta F508-WT15C (perfect) | 4416 | 591 |  | 4067 | 428 |  |
| 16) wt gDNA (500 pg) + delta F508-MUT15C (3 bp AAG del) | 5741 | 1144 | + 93.5 | 5509 | 960 | + 124 |

**Table S2.** Continued

| Sample | Fluorescence on Genexus argon laser @ PMT 34 after 30 min | TAF | % of difference relative to perfect match TAF | Fluorescence on Genexus argon laser @ PMT 34 after 45 min | TAF | % of difference relative to perfect match TAF |
| --- | --- | --- | --- | --- | --- | --- |
| 1) YOYO-1 (600 nM) | 187 |  |  | 207 |  |  |
| 2) delta F508-WT25C (3.2 pmole) (antisense) | 8116 |  |  | 7455 |  |  |
| 3) delta F508-MUT25C (3.2 pmole) (antisense) | 8363 |  |  | 8120 |  |  |
| 4) wt gDNA (500 pg) | 2802 |  |  | 2798 |  |  |
| 5) wt gDNA (500 pg) + delta F508-WT25C (perfect) | 17776 | 9660 |  | 17380 | 9925 |  |
| 6) wt gDNA (500 pg) + delta F508-MUT25C (3 bp AAG del) | 9092 | 729 | - 92.5 | 8953 | 833 | - 91.6 |
| 7) delta F508-WT20C (3.2 pmole) (antisense) | 5435 |  |  | 5193 |  |  |
| 8) delta F508-MUT20C (3.2 pmole) (antisense) | 826 |  |  | 802 |  |  |
| 9) wt gDNA (500 pg) | 2324 |  |  | 2300 |  |  |
| 10) wt gDNA (500 pg) + delta F508-WT20C (perfect) | 6652 | 1217 |  | 6549 | 1356 |  |
| 11) wt gDNA (500 pg) + delta F508-MUT20C (3 bp AAG del) | 997 | 171 | - 85.9 | 863 | 61 | - 95.5 |
| 12) delta F508-WT15C (3.2 pmole) (antisense) | 3528 |  |  | 3459 |  |  |
| 13) delta F508-MUT15C (3.2 pmole) (antisense) | 4398 |  |  | 5098 |  |  |
| 14) wt gDNA (500 pg) | 2534 |  |  | 2490 |  |  |
| 15) wt gDNA (500 pg) + delta F508-WT15C (perfect) | 4089 | 561 |  | 4139 | 680 |  |
| 16) wt gDNA (500 pg) + delta F508-MUT15C (3 bp AAG del) | 5384 | 986 | + 75.8 | 5213 | 115 | - 83.1 |

The target was human genomic dsDNA, wild-type for *CFTR*. The 15-mer probes were delta F508-WT15C (wild-type) and delta F508-MUT15C (mutant). The 20-mer probes were delta F508-WT20C (wild-type) and delta F508-MUT20C (mutant). The 25-mer probes were delta F508-WT25C (wild-type) and delta F508-MUT25C (mutant). 600 nM YOYO-1 was present in each sample. TAF indicates Triplex-Associated Fluorescence.
