# Supplementary material for: Heteropolymeric Triplex-Based Genomic Assay® to Detect Pathogens or Single-Nucleotide Polymorphisms in Human Genomic Samples
Source: PLoS One. 2007 Mar 21;2(3):e305. doi: 10.1371/journal.pone.0000305 (PMC1810429; doi:10.1371/journal.pone.0000305)
Supplement: Table S3. — Comparison of triplex assays of human genomic dsDNA using 20-mer, 25-mer or 30-mer ssDNA probes. Triplex assays of human genomic dsDNA for MTHFR C677T using 20-mer, 25-mer or 30-mer ssDNA probes demonstrate optimal probe length under the conditions employed, to be 25 bases. (0.06 MB DOC) [file pone.0000305.s009.doc]

# Table S3. Comparison of triplex assays of human genomic dsDNA using 20-mer, 25-mer or 30-mer ssDNA probes.

| Sample | Fluorescence on Genexus argon laser @ PMT 30 after 5 min | TAF | % of difference relative to perfect match TAF | Fluorescence on Genexus argon laser @ PMT 30 after 15 min | TAF | % of difference relative to perfect match TAF |
| --- | --- | --- | --- | --- | --- | --- |
| 1) YOYO-1 (500 nM) | 0 |  |  | 0 |  |  |
| 2) C677T-WT30C (3.2 pmole) (antisense) | 24304 |  |  | 22982 |  |  |
| 3) C677T-MUT30C (3.2 pmole) (antisense) | 1795 |  |  | 1537 |  |  |
| 4) wt gDNA (2 ng) | 2501 |  |  | 2234 |  |  |
| 5) wt gDNA (2 ng) + C677T-WT30C (perfect) | 38355 | 14051 |  | 37063 | 14081 |  |
| 6) wt gDNA (2 ng) + C677T-MUT30C (1 bp C-A) | 3138 | 1343 | - 90.4 | 2575 | 1038 | - 92.6 |
| 7) C677T-WT25C (3.2 pmole) (antisense) | 7856 |  |  | 7397 |  |  |
| 8) C677T-MUT25C (3.2 pmole) (antisense) | 1435 |  |  | 1241 |  |  |
| 9) wt gDNA (2 ng) | 2045 |  |  | 1596 |  |  |
| 10) wt gDNA (2 ng) + C677T-WT25C (perfect) | 31234 | 23378 |  | 30865 | 23468 |  |
| 11) wt gDNA (2 ng) + C677T-MUT25C (1 bp C-A) | 1882 | 447 | - 98.1 | 1474 | 233 | - 99.0 |
| 12) C677T-WT20C (3.2 pmole) (antisense) | 221 |  |  | 69 |  |  |
| 13) C677T-MUT20C (3.2 pmole) (antisense) | 3297 |  |  | 2639 |  |  |
| 14) wt gDNA (2 ng) | 2084 |  |  | 1883 |  |  |
| 15) wt gDNA (2 ng) + C677T-WT20C (perfect) | 1321 | 1100 |  | 854 | 785 |  |
| 16) wt gDNA (2 ng) + C677T-MUT20C (1 bp C-A) | 4245 | 948 | - 13.8 | 3083 | 444 | - 43.4 |

**Table S3.** Continued

| Sample | Fluorescence on Genexus argon laser @ PMT 30 after 30 min | TAF | % of difference relative to perfect match TAF | Fluorescence on Genexus argon laser @ PMT 30 after 45 min | TAF | % of difference relative to perfect match TAF |
| --- | --- | --- | --- | --- | --- | --- |
| 1) YOYO-1 (500 nM) | 0 |  |  | 0 |  |  |
| 2) C677T-WT30C (3.2 pmole) (antisense) | 22609 |  |  | 22302 |  |  |
| 3) C677T-MUT30C (3.2 pmole) (antisense) | 1304 |  |  | 1141 |  |  |
| 4) wt gDNA (2 ng) | 2162 |  |  | 2101 |  |  |
| 5) wt gDNA (2 ng) + C677T-WT30C (perfect) | 36679 | 14070 |  | 36279 | 13977 |  |
| 6) wt gDNA (2 ng) + C677T-MUT30C (1 bp C-A) | 2377 | 1073 | - 92.4 | 2078 | 937 | - 93.3 |
| 7) C677T-WT25C (3.2 pmole) (antisense) | 7060 |  |  | 6834 |  |  |
| 8) C677T-MUT25C (3.2 pmole) (antisense) | 1141 |  |  | 979 |  |  |
| 9) wt gDNA (2 ng) | 1518 |  |  | 1518 |  |  |
| 10) wt gDNA (2 ng) + C677T-WT25C (perfect) | 30392 | 23332 |  | 30697 | 23863 |  |
| 11) wt gDNA (2 ng) + C677T-MUT25C (1 bp C-A) | 1303 | 162 | - 99.3 | 1111 | 132 | - 99.4 |
| 12) C677T-WT20C (3.2 pmole) (antisense) | 24 |  |  | 4 |  |  |
| 13) C677T-MUT20C (3.2 pmole) (antisense) | 2345 |  |  | 2163 |  |  |
| 14) wt gDNA (2 ng) | 1788 |  |  | 1651 |  |  |
| 15) wt gDNA (2 ng) + C677T-WT20C (perfect) | 694 | 670 |  | 490 | 486 |  |
| 16) wt gDNA (2 ng) + C677T-MUT20C (1 bp C-A) | 2575 | 230 | - 65.7 | 2185 | 22 | - 95.5 |

The target was human genomic dsDNA, wild-type for *MTHFR*. The 20-mer probes were C677T-WT20C (wild-type) and C677T-MUT20C (mutant). The 25-mer probes were C677T-WT25C (wild-type) and C677T-MUT25C (mutant). The 30-mer probes were C677T-WT30C (wild-type) and C677T-MUT30C (mutant). 500 nM YOYO-1 was present in each sample. TAF indicates Triplex-Associated Fluorescence.
