# Supplementary material for: Heteropolymeric Triplex-Based Genomic Assay® to Detect Pathogens or Single-Nucleotide Polymorphisms in Human Genomic Samples
Source: PLoS One. 2007 Mar 21;2(3):e305. doi: 10.1371/journal.pone.0000305 (PMC1810429; doi:10.1371/journal.pone.0000305)
Supplement: Table S4. — Assays of varying concentrations of human genomic dsDNA for Factor V Leiden (1 bp G–T mismatch). The specificity of the triplex assay in detecting FVL G1691A in mismatched triplexes is demonstrated over a broad range of human genomic dsDNA concentrations. (0.07 MB DOC) [file pone.0000305.s010.doc]

**Table S4. Assays of varying concentrations of human genomic dsDNA for Factor V Leiden (1 bp G-T mismatch).**

| Sample | Fluorescence on Genexus argon laser @ PMT 30 after 5 min | TAF | % of difference relative to perfect match TAF | Fluorescence on Genexus argon laser @ PMT 30 after 15 min | TAF | % of difference relative to perfect match TAF |
| --- | --- | --- | --- | --- | --- | --- |
| 1) YOYO-1 (500 nM) | 0 |  |  | 0 |  |  |
| 2) FVL-WT25C (3.2 pmole) (antisense) | 6206 |  |  | 6035 |  |  |
| 3) FVL-MUT25C (3.2 pmole) (antisense) | 44456 |  |  | 44427 |  |  |
| 4) wt gDNA (2 ng) | 3409 |  |  | 3421 |  |  |
| 5) wt gDNA (2 ng) + FVL-WT25C (perfect) | 26341 | 20135 |  | 26355 | 20320 |  |
| 6) wt gDNA (2 ng) +FVL-MUT25C (1 bp G-T) | 34222 | < 0 | - 100 | 33941 | < 0 | - 100 |
| 7) wt gDNA (1 ng) | 1198 |  |  | 1348 |  |  |
| 8) wt gDNA (1 ng) + FVL-WT25C (perfect) | 20409 | 14203 |  | 21450 | 15415 |  |
| 9) wt gDNA (1 ng) + FVL-MUT25C (1 bp G-T) | 25049 | < 0 | - 100 | 24984 | < 0 | - 100 |
| 10) wt gDNA (500 pg) | 4 |  |  | 6 |  |  |
| 11) wt gDNA (500 pg) + FVL-WT25C (perfect) | 23757 | 17551 |  | 23451 | 17416 |  |
| 12) wt gDNA (500 pg) + FVL-MUT25C (1 bp G-T) | 25594 | < 0 | - 100 | 25480 | < 0 | - 100 |
| 13) wt gDNA (200 pg) | 7 |  |  | 0 |  |  |
| 14) wt gDNA (200 pg) + FVL-WT25C (perfect) | 20925 | 14719 |  | 20874 | 14839 |  |
| 15) wt gDNA (200 pg) + FVL-MUT25C (1 bp G-T) | 22325 | < 0 | - 100 | 22378 | < 0 | - 100 |

**Table S4.** Continued

| Sample | Fluorescence on Genexus argon laser @ PMT 30 after 30 min | TAF | % of difference relative to perfect match TAF | Fluorescence on Genexus argon laser @ PMT 30 after 45 min | TAF | % of difference relative to perfect match TAF |
| --- | --- | --- | --- | --- | --- | --- |
| 1) YOYO-1 (500 nM) | 0 |  |  | 0 |  |  |
| 2) FVL-WT25C (3.2 pmole) (antisense) | 5803 |  |  | 5518 |  |  |
| 3) FVL-MUT25C (3.2 pmole) (antisense) | 44650 |  |  | 44101 |  |  |
| 4) wt gDNA (2 ng) | 3687 |  |  | 3642 |  |  |
| 5) wt gDNA (2 ng) + FVL-WT25C (perfect) | 26378 | 20575 |  | 26041 | 20523 |  |
| 6) wt gDNA (2 ng) +FVL-MUT25C (1 bp G-T) | 34069 | < 0 | - 100 | 33633 | < 0 | - 100 |
| 7) wt gDNA (1 ng) | 1474 |  |  | 1598 |  |  |
| 8) wt gDNA (1 ng) + FVL-WT25C (perfect) | 22017 | 16214 |  | 21862 | 16344 |  |
| 9) wt gDNA (1 ng) + FVL-MUT25C (1 bp G-T) | 25098 | < 0 | - 100 | 24793 | < 0 | - 100 |
| 10) wt gDNA (500 pg) | 8 |  |  | 14 |  |  |
| 11) wt gDNA (500 pg) + FVL-WT25C (perfect) | 23417 | 17614 |  | 23159 | 17641 |  |
| 12) wt gDNA (500 pg) + FVL-MUT25C (1 bp G-T) | 25514 | < 0 | - 100 | 25347 | < 0 | - 100 |
| 13) wt gDNA (200 pg) | 0 |  |  | 0 |  |  |
| 14) wt gDNA (200 pg) + FVL-WT25C (perfect) | 20852 | 15049 |  | 20698 | 15180 |  |
| 15) wt gDNA (200 pg) + FVL-MUT25C (1 bp G-T) | 22441 | < 0 | - 100 | 22353 | < 0 | - 100 |

**Table S4.** Continued

| Sample | Fluorescence on Genexus argon laser @ PMT 30 after 60 min | TAF | % of difference relative to perfect match TAF |
| --- | --- | --- | --- |
| 1) YOYO-1 (500 nM) | 0 |  |  |
| 2) FVL-WT25C (3.2 pmole) (antisense) | 5505 |  |  |
| 3) FVL-MUT25C (3.2 pmole) (antisense) | 44534 |  |  |
| 4) wt gDNA (2 ng) | 3619 |  |  |
| 5) wt gDNA (2 ng) + FVL-WT25C (perfect) | 26024 | 20519 |  |
| 6) wt gDNA (2 ng) +FVL-MUT25C (1 bp G-T) | 33801 | < 0 | - 100 |
| 7) wt gDNA (1 ng) | 1643 |  |  |
| 8) wt gDNA (1 ng) + FVL-WT25C (perfect) | 22005 | 16500 |  |
| 9) wt gDNA (1 ng) + FVL-MUT25C (1 bp G-T) | 24913 | < 0 | - 100 |
| 10) wt gDNA (500 pg) | 16 |  |  |
| 11) wt gDNA (500 pg) + FVL-WT25C (perfect) | 23240 | 17735 |  |
| 12) wt gDNA (500 pg) + FVL-MUT25C (1 bp G-T) | 25483 | < 0 | - 100 |
| 13) wt gDNA (200 pg) | 0 |  |  |
| 14) wt gDNA (200 pg) + FVL-WT25C (perfect) | 20720 | 15215 |  |
| 15) wt gDNA (200 pg) + FVL-MUT25C (1 bp G-T) | 22448 | < 0 | - 100 |

The target was human genomic dsDNA, wild-type for *FVL*. The 25-mer probes were FVL-WT25C (wild-type) and FVL-MUT25C (mutant). 500 nM YOYO-1 was present in each sample. TAF indicates Triplex-Associated Fluorescence.
