# Supplementary material for: Heteropolymeric Triplex-Based Genomic Assay® to Detect Pathogens or Single-Nucleotide Polymorphisms in Human Genomic Samples
Source: PLoS One. 2007 Mar 21;2(3):e305. doi: 10.1371/journal.pone.0000305 (PMC1810429; doi:10.1371/journal.pone.0000305)
Supplement: Table S5. — Assays of varying concentrations of human genomic dsDNA for CFTR 2789+5G→A (1 bp T–G mismatch). The specificity of the triplex assay in detecting CFTR 2789+5G→A in mismatched triplexes is demonstrated over a broad range of human genomic dsDNA concentrations. (0.08 MB DOC) [file pone.0000305.s011.doc]

**Table S5. Assays of varying concentrations of human genomic dsDNA for *CFTR* 2789+5G->A (1 bp T-G mismatch).**

| Sample | Fluorescence on Genexus argon laser @ PMT 30 after 5 min | TAF | % of difference relative to perfect match TAF | Fluorescence on Genexus argon laser @ PMT 30 after 15 min | TAF | % of difference relative to perfect match TAF |
| --- | --- | --- | --- | --- | --- | --- |
| 1) YOYO-1 (500 nM) | 32 |  |  | 27 |  |  |
| 2) 2789+5G->A-WT25C (3.2 pmole) (antisense) | 10153 |  |  | 8203 |  |  |
| 3) 2789+5G->A-MUT25C (3.2 pmole) (antisense) | 14032 |  |  | 14241 |  |  |
| 4) wt gDNA (500 pg) | 11 |  |  | 41 |  |  |
| 5) wt gDNA (500 pg) + 2789+5G->A-WT25C (perfect) | 29062 | 18909 |  | 29781 | 21578 |  |
| 6) wt gDNA (500 pg) + 2789+5G->A-MUT25C (1 bp T-G) | 11601 | < 0 | - 100 | 11654 | < 0 | - 100 |
| 7) wt gDNA (200 pg) | 0 |  |  | 0 |  |  |
| 8) wt gDNA (200 pg) + 2789+5G->A-WT25C (perfect) | 25625 | 15472 |  | 25912 | 17409 |  |
| 9) wt gDNA (200 pg) + 2789+5G->A-MUT25C (1 bp T-G) | 11068 | < 0 | - 100 | 10857 | < 0 | - 100 |
| 10) wt gDNA (100 pg) | 0 |  |  | 0 |  |  |
| 11) wt gDNA (100 pg) + 2789+5G->A-WT25C (perfect) | 23859 | 13706 |  | 23734 | 15531 |  |
| 12) wt gDNA (100 pg) + 2789+5G->A-MUT25C (1 bp T-G) | 11442 | < 0 | - 100 | 11198 | < 0 | - 100 |
| 13) wt gDNA (75 pg) | 0 |  |  | 0 |  |  |
| 14) wt gDNA (75 pg) + 2789+5G->A-WT25C (perfect) | 18318 | 8165 |  | 17576 | 9373 |  |
| 15) wt gDNA (75 pg) + 2789+5G->A-MUT25C (1 bp T-G) | 9684 | < 0 | - 100 | 9395 | < 0 | - 100 |

**Table S5.** Continued

| Sample | Fluorescence on Genexus argon laser @ PMT 30 after 30 min | TAF | % of difference relative to perfect match TAF | Fluorescence on Genexus argon laser @ PMT 30 after 45 min | TAF | % of difference relative to perfect match TAF |
| --- | --- | --- | --- | --- | --- | --- |
| 1) YOYO-1 (500 nM) | 32 |  |  | 47 |  |  |
| 2) 2789+5G->A-WT25C (3.2 pmole) (antisense) | 8007 |  |  | 8095 |  |  |
| 3) 2789+5G->A-MUT25C (3.2 pmole) (antisense) | 14190 |  |  | 14033 |  |  |
| 4) wt gDNA (500 pg) | 14 |  |  | 6 |  |  |
| 5) wt gDNA (500 pg) + 2789+5G->A-WT25C (perfect) | 29230 | 21223 |  | 28837 | 20742 |  |
| 6) wt gDNA (500 pg) + 2789+5G->A-MUT25C (1 bp T-G) | 11613 | < 0 | - 100 | 11489 | < 0 | - 100 |
| 7) wt gDNA (200 pg) | 0 |  |  | 0 |  |  |
| 8) wt gDNA (200 pg) + 2789+5G->A-WT25C (perfect) | 24879 | 16872 |  | 24435 | 16340 |  |
| 9) wt gDNA (200 pg) + 2789+5G->A-MUT25C (1 bp T-G) | 10299 | < 0 | - 100 | 10244 | < 0 | - 100 |
| 10) wt gDNA (100 pg) | 0 |  |  | 0 |  |  |
| 11) wt gDNA (100 pg) + 2789+5G->A-WT25C (perfect) | 23530 | 15523 |  | 23707 | 15612 |  |
| 12) wt gDNA (100 pg) + 2789+5G->A-MUT25C (1 bp T-G) | 10924 | < 0 | - 100 | 10749 | < 0 | - 100 |
| 13) wt gDNA (75 pg) | 0 |  |  | 0 |  |  |
| 14) wt gDNA (75 pg) + 2789+5G->A-WT25C (perfect) | 17659 | 9652 |  | 17874 | 9779 |  |
| 15) wt gDNA (75 pg) + 2789+5G->A-MUT25C (1 bp T-G) | 9091 | < 0 | - 100 | 9287 | < 0 | - 100 |

**Table S5.** Continued

| Sample | Fluorescence on Genexus argon laser @ PMT 30 after 60 min | TAF | % of difference relative to perfect match TAF | Fluorescence on Genexus argon laser @ PMT 30 after 75 min | TAF | % of difference relative to perfect match TAF |
| --- | --- | --- | --- | --- | --- | --- |
| 1) YOYO-1 (500 nM) | 31 |  |  | 53 |  |  |
| 2) 2789+5G->A-WT25C (3.2 pmole) (antisense) | 8232 |  |  | 8290 |  |  |
| 3) 2789+5G->A-MUT25C (3.2 pmole) (antisense) | 13916 |  |  | 13843 |  |  |
| 4) wt gDNA (500 pg) | 2 |  |  | 50 |  |  |
| 5) wt gDNA (500 pg) + 2789+5G->A-WT25C (perfect) | 28606 | 20374 |  | 28563 | 20273 |  |
| 6) wt gDNA (500 pg) + 2789+5G->A-MUT25C (1 bp T-G) | 11313 | < 0 | - 100 | 11240 | < 0 | - 100 |
| 7) wt gDNA (200 pg) | 0 |  |  | 0 |  |  |
| 8) wt gDNA (200 pg) + 2789+5G->A-WT25C (perfect) | 23858 | 15626 |  | 23513 | 15223 |  |
| 9) wt gDNA (200 pg) + 2789+5G->A-MUT25C (1 bp T-G) | 10068 | < 0 | - 100 | 10020 | < 0 | - 100 |
| 10) wt gDNA (100 pg) | 0 |  |  | 0 |  |  |
| 11) wt gDNA (100 pg) + 2789+5G->A-WT25C (perfect) | 23846 | 15614 |  | 23720 | 15430 |  |
| 12) wt gDNA (100 pg) + 2789+5G->A-MUT25C (1 bp T-G) | 10593 | < 0 | - 100 | 10527 | < 0 | - 100 |
| 13) wt gDNA (75 pg) | 0 |  |  | 0 |  |  |
| 14) wt gDNA (75 pg) + 2789+5G->A-WT25C (perfect) | 17573 | 9341 |  | 17506 | 9216 |  |
| 15) wt gDNA (75 pg) + 2789+5G->A-MUT25C (1 bp T-G) | 9054 | < 0 | - 100 | 9070 | < 0 | - 100 |

# The target was human genomic dsDNA, wild-type for *CFTR*. The 25-mer probes were 2789+5G->A-WT25C (wild-type) and 2789+5G->A-MUT25C (mutant). 500 nM YOYO-1 was present in each sample. TAF indicates Triplex-Associated Fluorescence.
