# Supplementary material for: Heteropolymeric Triplex-Based Genomic Assay® to Detect Pathogens or Single-Nucleotide Polymorphisms in Human Genomic Samples
Source: PLoS One. 2007 Mar 21;2(3):e305. doi: 10.1371/journal.pone.0000305 (PMC1810429; doi:10.1371/journal.pone.0000305)
Supplement: Table S6. — Assays of human genomic dsDNA (purified from blood) for CFTR 2789+5G→A (1 bp T–G mismatch). The specificity of the triplex assay in detecting CFTR 2789+5G→A in mismatched triplexes comprising human genomic dsDNA purified from blood is demonstrated. (0.04 MB DOC) [file pone.0000305.s012.doc]

**Table S6. Assays of human genomic dsDNA (purified from blood) for *CFTR* 2789+5G->A (1 bp T-G mismatch).**

| Sample | Fluorescence on Genexus argon laser @ PMT 30 after 5 min | TAF | % of difference relative to perfect match TAF | Fluorescence on Genexus argon laser @ PMT 30 after 15 min | TAF | % of difference relative to perfect match TAF |
| --- | --- | --- | --- | --- | --- | --- |
| 1) YOYO-1 (500 nM) | 0 |  |  | 0 |  |  |
| 2) 2789+5G->A-WT25C (3.2 pmole) (antisense) | 22277 |  |  | 25513 |  |  |
| 3) 2789+5G->A-MUT25C (3.2 pmole) (antisense) | 18238 |  |  | 17386 |  |  |
| 4) wt gDNA (4 ng) | 6613 |  |  | 6529 |  |  |
| 5) wt gDNA (4 ng) + 2789+5G->A-WT25C (perfect) | 40478 | 18201 |  | 39159 | 13646 |  |
| 6) wt gDNA (4 ng) + 2789+5G->A-MUT25C (1 bp T-G) | 15394 | < 0 | - 100 | 13388 | < 0 | - 100 |
| 7) wt gDNA (2 ng) | 2925 |  |  | 2819 |  |  |
| 8) wt gDNA (2 ng) + 2789+5G->A-WT25 (perfect) | 39407 | 17130 |  | 38648 | 13135 |  |
| 9) wt gDNA (2 ng) + 2789+5G->A-MUT25 (1 bp T-G) | 20647 | 2409 | - 85.9 | 20329 | 2943 | - 77.6 |

**Table S6.** Continued

| Sample | Fluorescence on Genexus argon laser @ PMT 30 after 30 min | TAF | % of difference relative to perfect match TAF |
| --- | --- | --- | --- |
| 1) YOYO-1 (500 nM) | 0 |  |  |
| 2) 2789+5G->A-WT25C (3.2 pmole) (antisense) | 25795 |  |  |
| 3) 2789+5G->A-MUT25C (3.2 pmole) (antisense) | 17343 |  |  |
| 4) wt gDNA (4 ng) | 6504 |  |  |
| 5) wt gDNA (4 ng) + 2789+5G->A-WT25C (perfect) | 39626 | 13831 |  |
| 6) wt gDNA (4 ng) + 2789+5G->A-MUT25C (1 bp T-G) | 12248 | < 0 | - 100 |
| 7) wt gDNA (2 ng) | 2855 |  |  |
| 8) wt gDNA (2 ng) + 2789+5G->A-WT25 (perfect) | 38834 | 13039 |  |
| 9) wt gDNA (2 ng) + 2789+5G->A-MUT25 (1 bp T-G) | 19984 | 2641 | - 79.7 |

The target was human genomic dsDNA, wild-type for *CFTR*. The 25-mer probes were 2789+5G->A-WT25C (wild-type) and 2789+5G->A-MUT25C (mutant). 500 nM YOYO-1 was present in each sample. TAF indicates Triplex-Associated Fluorescence.
