# Supplementary material for: Heteropolymeric Triplex-Based Genomic Assay® to Detect Pathogens or Single-Nucleotide Polymorphisms in Human Genomic Samples
Source: PLoS One. 2007 Mar 21;2(3):e305. doi: 10.1371/journal.pone.0000305 (PMC1810429; doi:10.1371/journal.pone.0000305)
Supplement: Table S7. — Assays of human genomic dsDNA (wild-type homozygous or mutant heterozygous samples purified from blood) for MTHFR C677T. The specificity of the triplex assay in assaying wild-type homozygous and mutant heterozygous human genomic dsDNA samples for MTHFR C677T is demonstrated. (0.06 MB DOC) [file pone.0000305.s013.doc]

# Table S7. Assays of human genomic dsDNA (wild-type homozygous or mutant heterozygous samples purified from blood) for *MTHFR* C677T.

| Sample | Fluorescence on Genexus argon laser @ PMT 30 after 5 min | TAF | % of difference relative to perfect match TAF | Fluorescence on Genexus argon laser @ PMT 30 after 15 min | TAF | % of difference relative to perfect match TAF |
| --- | --- | --- | --- | --- | --- | --- |
| 1) YOYO-1 (500 nM) | 0 |  |  | 0 |  |  |
| 2) MTHFR-WT25C (3.2 pmole) (antisense) | 4939 |  |  | 4362 |  |  |
| 3) MTHFR-MUT25C (3.2 pmole) (antisense) | 963 |  |  | 842 |  |  |
| 4) wt gDNA (2 ng) | 3558 |  |  | 3530 |  |  |
| 5) mut hetero gDNA (2 ng) | 6226 |  |  | 6047 |  |  |
| 6) wt gDNA (2 ng) + MTHFR-WT25C (perfect) | 13544 | 9151 |  | 13041 | 8679 |  |
| 7) wt gDNA (2 ng) + MTHFR-MUT25C (1 bp C-A) | 2808 | 1845 | - 79.8 | 2419 | 1577 | - 81.8 |
| 8) mut hetero gDNA (2 ng) + MTHFR-MUT25C (hetero) | 4357 | 3394 | - 62.9 | 3840 | 2998 | - 65.5 |
| 9) wt gDNA (1 ng) | 1122 |  |  | 1112 |  |  |
| 10) mut hetero gDNA (1 ng) | 2246 |  |  | 2229 |  |  |
| 11) wt gDNA (1 ng) + MTHFR-WT25C (perfect) | 10823 | 5884 |  | 10331 | 5969 |  |
| 12) wt gDNA (1 ng) + MTHFR-MUT25C (1 bp C-A) | 1438 | 475 | - 91.9 | 1159 | 317 | - 94.7 |
| 13) mut hetero gDNA (1 ng) + MTHFR-MUT25C (hetero) | 2343 | 1380 | - 76.5 | 2045 | 1203 | - 79.8 |

**Table S7.** Continued

| Sample | Fluorescence on Genexus argon laser @ PMT 30 after 30 min | TAF | % of difference relative to perfect match TAF | Fluorescence on Genexus argon laser @ PMT 30 after 45 min | TAF | % of difference relative to perfect match TAF |
| --- | --- | --- | --- | --- | --- | --- |
| 1) YOYO-1 (500 nM) | 0 |  |  | 0 |  |  |
| 2) MTHFR-WT25C (3.2 pmole) (antisense) | 3980 |  |  | 3773 |  |  |
| 3) MTHFR-MUT25C (3.2 pmole) (antisense) | 780 |  |  | 717 |  |  |
| 4) wt gDNA (2 ng) | 3472 |  |  | 3395 |  |  |
| 5) mut hetero gDNA (2 ng) | 5843 |  |  | 5648 |  |  |
| 6) wt gDNA (2 ng) + MTHFR-WT25C (perfect) | 12659 | 8679 |  | 12315 | 8542 |  |
| 7) wt gDNA (2 ng) + MTHFR-MUT25C (1 bp C-A) | 2073 | 1293 | - 85.1 | 1855 | 1138 | - 86.7 |
| 8) mut hetero gDNA (2 ng) + MTHFR-MUT25C (hetero) | 3294 | 2514 | - 71.0 | 2888 | 2171 | - 74.6 |
| 9) wt gDNA (1 ng) | 1076 |  |  | 1032 |  |  |
| 10) mut hetero gDNA (1 ng) | 2123 |  |  | 2003 |  |  |
| 11) wt gDNA (1 ng) + MTHFR-WT25C (perfect) | 9914 | 5934 |  | 9531 | 5758 |  |
| 12) wt gDNA (1 ng) + MTHFR-MUT25C (1 bp C-A) | 926 | 146 | - 97.5 | 833 | 116 | - 98.0 |
| 13) mut hetero gDNA (1 ng) + MTHFR-MUT25C (hetero) | 1687 | 907 | - 84.7 | 1383 | 666 | - 88.4 |

**Table S7.** Continued

| Sample | Fluorescence on Genexus argon laser @ PMT 30 after 60 min | TAF | % of difference relative to perfect match TAF |
| --- | --- | --- | --- |
| 1) YOYO-1 (500 nM) | 0 |  |  |
| 2) MTHFR-WT25C (3.2 pmole) (antisense) | 3588 |  |  |
| 3) MTHFR-MUT25C (3.2 pmole) (antisense) | 626 |  |  |
| 4) wt gDNA (2 ng) | 3355 |  |  |
| 5) mut hetero gDNA (2 ng) | 5599 |  |  |
| 6) wt gDNA (2 ng) + MTHFR-WT25C (perfect) | 12177 | 8589 |  |
| 7) wt gDNA (2 ng) + MTHFR-MUT25C (1 bp C-A) | 1754 | 1128 | - 86.9 |
| 8) mut hetero gDNA (2 ng) + MTHFR-MUT25C (hetero) | 2723 | 2097 | - 75.6 |
| 9) wt gDNA (1 ng) | 1014 |  |  |
| 10) mut hetero gDNA (1 ng) | 1944 |  |  |
| 11) wt gDNA (1 ng) + MTHFR-WT25C (perfect) | 9341 | 5753 |  |
| 12) wt gDNA (1 ng) + MTHFR-MUT25C (1 bp C-A) | 721 | 95 | - 98.3 |
| 13) mut hetero gDNA (1 ng) + MTHFR-MUT25C (hetero) | 1222 | 596 | - 89.6 |

The target was human genomic dsDNA, wild-type homozygous for *MTHFR* or human genomic dsDNA, mutant heterozygous for *MTHFR*. The 25-mer probes were MTHFR-WT25C (wild-type) and MTHFR-MUT25C (mutant). 500 nM YOYO-1 was present in each sample. TAF indicates Triplex-Associated Fluorescence.
