# Supplementary material for: Heteropolymeric Triplex-Based Genomic Assay® to Detect Pathogens or Single-Nucleotide Polymorphisms in Human Genomic Samples
Source: PLoS One. 2007 Mar 21;2(3):e305. doi: 10.1371/journal.pone.0000305 (PMC1810429; doi:10.1371/journal.pone.0000305)
Supplement: Table S8. — Assays of human genomic dsDNA (mutant homozygous sample purified from blood) for MTHFR C677T. The specificity of the triplex assay in assaying mutant homozygous human genomic dsDNA samples for MTHFR C677T is demonstrated. (0.05 MB DOC) [file pone.0000305.s014.doc]

# Table S8. Assays of human genomic dsDNA (mutant homozygous sample purified from blood) for *MTHFR* C677T.

| Sample | Fluorescence on Genexus argon laser @ PMT 30 after 5 min | TAF | % of difference relative to perfect match TAF | Fluorescence on Genexus argon laser @ PMT 30 after 15 min | TAF | % of difference relative to perfect match TAF |
| --- | --- | --- | --- | --- | --- | --- |
| 1) YOYO-1 (500 nM) | 0 |  |  | 0 |  |  |
| 2) MTHFR-WT25C (3.2 pmole) (antisense) | 4523 |  |  | 4452 |  |  |
| 3) MTHFR-MUT25C (3.2 pmole) (antisense) | 748 |  |  | 597 |  |  |
| 4) mutant homozygous gDNA (2 ng) | 3276 |  |  | 3245 |  |  |
| 5) mut homo gDNA (2 ng) + MTHFR-MUT25C (perfect) | 10619 | 9871 |  | 10223 | 9626 |  |
| 6) mut homo gDNA (2 ng) + MTHFR-WT25C (1 bp C-A) | 2041 | < 0 | - 100 | 1653 | < 0 | - 100 |
| 7) mut homo gDNA (1 ng) | 1048 |  |  | 1037 |  |  |
| 8) mut homo gDNA (1 ng) + MTHFR-MUT25C (perfect) | 8343 | 7595 |  | 7598 | 7001 |  |
| 9) mut homo gDNA (1 ng) + MTHFR-WT25C (1 bp C-A) | 1356 | < 0 | - 100 | 1091 | < 0 | - 100 |

**Table S8.** Continued

| Sample | Fluorescence on Genexus argon laser @ PMT 30 after 30 min | TAF | % of difference relative to perfect match TAF | Fluorescence on Genexus argon laser @ PMT 30 after 45 min | TAF | % of difference relative to perfect match TAF |
| --- | --- | --- | --- | --- | --- | --- |
| 1) YOYO-1 (500 nM) | 0 |  |  | 0 |  |  |
| 2) MTHFR-WT25C (3.2 pmole) (antisense) | 4744 |  |  | 4954 |  |  |
| 3) MTHFR-MUT25C (3.2 pmole) (antisense) | 530 |  |  | 427 |  |  |
| 4) mutant homozygous gDNA (2 ng) | 3041 |  |  | 2809 |  |  |
| 5) mut homo gDNA (2 ng) + MTHFR-MUT25C (perfect) | 9828 | 9298 |  | 9593 | 9166 |  |
| 6) mut homo gDNA (2 ng) + MTHFR-WT25C (1 bp C-A) | 1297 | < 0 | - 100 | 1131 | < 0 | - 100 |
| 7) mut homo gDNA (1 ng) | 927 |  |  | 796 |  |  |
| 8) mut homo gDNA (1 ng) + MTHFR-MUT25C (perfect) | 7182 | 6652 |  | 6896 | 6469 |  |
| 9) mut homo gDNA (1 ng) + MTHFR-WT25C (1 bp C-A) | 880 | < 0 | - 100 | 818 | < 0 | - 100 |

**Table S8.** Continued

| Sample | Fluorescence on Genexus argon laser @ PMT 30 after 60 min | TAF | % of difference relative to perfect match TAF |
| --- | --- | --- | --- |
| 1) YOYO-1 (500 nM) | 0 |  |  |
| 2) MTHFR-WT25C (3.2 pmole) (antisense) | 4937 |  |  |
| 3) MTHFR-MUT25C (3.2 pmole) (antisense) | 340 |  |  |
| 4) mutant homozygous gDNA (2 ng) | 2786 |  |  |
| 5) mut homo gDNA (2 ng) + MTHFR-MUT25C (perfect) | 9387 | 9047 |  |
| 6) mut homo gDNA (2 ng) + MTHFR-WT25C (1 bp C-A) | 1014 | < 0 | - 100 |
| 7) mut homo gDNA (1 ng) | 763 |  |  |
| 8) mut homo gDNA (1 ng) + MTHFR-MUT25C (perfect) | 6632 | 6292 |  |
| 9) mut homo gDNA (1 ng) + MTHFR-WT25C (1 bp C-A) | 689 | < 0 | - 100 |

The target was human genomic dsDNA, mutant homozygous for *MTHFR*. The 25-mer probes were MTHFR-WT25C (wild-type) and MTHFR-MUT25C (mutant). 500 nM YOYO-1 was present in each sample. TAF indicates Triplex-Associated Fluorescence.
