# Supplementary material for: Heteropolymeric Triplex-Based Genomic Assay® to Detect Pathogens or Single-Nucleotide Polymorphisms in Human Genomic Samples
Source: PLoS One. 2007 Mar 21;2(3):e305. doi: 10.1371/journal.pone.0000305 (PMC1810429; doi:10.1371/journal.pone.0000305)
Supplement: Table S9. — Assays of varying concentrations of human genomic dsDNA for MTHFR C677T (1 bp C–A mismatch). The specificity of the triplex assay in detecting MTHFR C677T in mismatched triplexes is demonstrated over a broad range of human genomic dsDNA concentrations. (0.07 MB DOC) [file pone.0000305.s015.doc]

**Table S9. Assays of varying concentrations of human genomic dsDNA for *MTHFR* C677T (1 bp C-A mismatch).**

| Sample | Fluorescence on Genexus argon laser @ PMT 30 after 5 min | TAF | % of difference relative to perfect match TAF | Fluorescence on Genexus argon laser @ PMT 30 after 15 min | TAF | % of difference relative to perfect match TAF |
| --- | --- | --- | --- | --- | --- | --- |
| 1) YOYO-1 (500 nM) | 0 |  |  | 0 |  |  |
| 2) MTHFR-WT25C (3.2 pmole) (antisense) | 4498 |  |  | 3987 |  |  |
| 3) MTHFR-MUT25C (3.2 pmole) (antisense) | 882 |  |  | 795 |  |  |
| 4) wt gDNA (2 ng) | 2614 |  |  | 2573 |  |  |
| 5) wt gDNA (2 ng) + MTHFR-WT25C (perfect) | 11989 | 7491 |  | 11418 | 7431 |  |
| 6) wt gDNA (2 ng) + MTHFR-MUT25C (1 bp C-A) | 1698 | 816 | - 89.1 | 1509 | 714 | - 90.4 |
| 7) wt gDNA (1 ng) | 1005 |  |  | 1174 |  |  |
| 8) wt gDNA (1 ng) + MTHFR-WT25C (perfect) | 9963 | 5465 |  | 9366 | 5379 |  |
| 9) wt gDNA (1 ng) + MTHFR-MUT25C (1 bp C-A) | 1179 | 297 | - 94.6 | 1066 | 271 | - 95.0 |
| 10) wt gDNA (500 pg) | 0 |  |  | 0 |  |  |
| 11) wt gDNA (500 pg) + MTHFR-WT25C (perfect) | 10088 | 5590 |  | 9628 | 5641 |  |
| 12) wt gDNA (500 pg) + MTHFR-MUT25C (1 bp C-A) | 726 | < 0 | - 100 | 670 | < 0 | - 100 |
| 13) wt gDNA (200 pg) | 0 |  |  | 0 |  |  |
| 14) wt gDNA (200 pg) + MTHFR-WT25C (perfect) | 5628 | 1130 |  | 5021 | 1034 |  |
| 15) wt gDNA (200 pg) + MTHFR-MUT25C (1 bp C-A) | 918 | 36 | - 94.3 | 783 | < 0 | - 100 |

**Table S9.** Continued

| Sample | Fluorescence on Genexus argon laser @ PMT 30 after 30 min | TAF | % of difference relative to perfect match TAF | Fluorescence on Genexus argon laser @ PMT 30 after 45 min | TAF | % of difference relative to perfect match TAF |
| --- | --- | --- | --- | --- | --- | --- |
| 1) YOYO-1 (500 nM) | 0 |  |  | 0 |  |  |
| 2) MTHFR-WT25C (3.2 pmole) (antisense) | 3877 |  |  | 3695 |  |  |
| 3) MTHFR-MUT25C (3.2 pmole) (antisense) | 792 |  |  | 633 |  |  |
| 4) wt gDNA (2 ng) | 2524 |  |  | 2484 |  |  |
| 5) wt gDNA (2 ng) + MTHFR-WT25C (perfect) | 11412 | 7535 |  | 11161 | 7466 |  |
| 6) wt gDNA (2 ng) + MTHFR-MUT25C (1 bp C-A) | 1348 | 556 | - 92.6 | 1203 | 570 | - 92.4 |
| 7) wt gDNA (1 ng) | 1514 |  |  | 1679 |  |  |
| 8) wt gDNA (1 ng) + MTHFR-WT25C (perfect) | 9035 | 5158 |  | 9000 | 5305 |  |
| 9) wt gDNA (1 ng) + MTHFR-MUT25C (1 bp C-A) | 922 | 130 | - 97.5 | 806 | 173 | - 96.7 |
| 10) wt gDNA (500 pg) | 0 |  |  | 0 |  |  |
| 11) wt gDNA (500 pg) + MTHFR-WT25C (perfect) | 9272 | 5395 |  | 9172 | 5477 |  |
| 12) wt gDNA (500 pg) + MTHFR-MUT25C (1 bp C-A) | 640 | < 0 | - 100 | 509 | < 0 | - 100 |
| 13) wt gDNA (200 pg) | 0 |  |  | 9 |  |  |
| 14) wt gDNA (200 pg) + MTHFR-WT25C (perfect) | 4612 | 735 |  | 4309 | 614 |  |
| 15) wt gDNA (200 pg) + MTHFR-MUT25C (1 bp C-A) | 673 | < 0 | - 100 | 542 | < 0 | - 100 |

**Table S9.** Continued

| Sample | Fluorescence on Genexus argon laser @ PMT 30 after 60 min | TAF | % of difference relative to perfect match TAF |
| --- | --- | --- | --- |
| 1) YOYO-1 (500 nM) | 0 |  |  |
| 2) MTHFR-WT25C (3.2 pmole) (antisense) | 3439 |  |  |
| 3) MTHFR-MUT25C (3.2 pmole) (antisense) | 515 |  |  |
| 4) wt gDNA (2 ng) | 2445 |  |  |
| 5) wt gDNA (2 ng) + MTHFR-WT25C (perfect) | 10802 | 7363 |  |
| 6) wt gDNA (2 ng) + MTHFR-MUT25C (1 bp C-A) | 643 | 128 | - 98.3 |
| 7) wt gDNA (1 ng) | 367 |  |  |
| 8) wt gDNA (1 ng) + MTHFR-WT25C (perfect) | 8660 | 5221 |  |
| 9) wt gDNA (1 ng) + MTHFR-MUT25C (1 bp C-A) | 710 | 195 | - 96.3 |
| 10) wt gDNA (500 pg) | 0 |  |  |
| 11) wt gDNA (500 pg) + MTHFR-WT25C (perfect) | 8907 | 5468 |  |
| 12) wt gDNA (500 pg) + MTHFR-MUT25C (1 bp C-A) | 849 | 334 | - 93.9 |
| 13) wt gDNA (200 pg) | 0 |  |  |
| 14) wt gDNA (200 pg) + MTHFR-WT25C (perfect) | 4131 | 692 |  |
| 15) wt gDNA (200 pg) + MTHFR-MUT25C (1 bp C-A) | 257 | < 0 | - 100 |

The target was human genomic dsDNA, wild-type for *MTHFR*. The 25-mer probes were MTHFR-WT25C (wild-type) and MTHFR-MUT25C (mutant). 500 nM YOYO-1 was present in each sample. TAF indicates Triplex-Associated Fluorescence.
