# Supplementary material for: Heteropolymeric Triplex-Based Genomic Assay® to Detect Pathogens or Single-Nucleotide Polymorphisms in Human Genomic Samples
Source: PLoS One. 2007 Mar 21;2(3):e305. doi: 10.1371/journal.pone.0000305 (PMC1810429; doi:10.1371/journal.pone.0000305)
Supplement: Table S10. — Assays of human genomic dsDNA employing varying protocols. The specificity of the triplex assay in detecting CFTR 3849+10kbC→T in mismatched triplexes is demonstrated in reaction mixtures containing a kosmotropic agent. Two reaction protocols are compared. (0.05 MB DOC) [file pone.0000305.s016.doc]

**Table S10. Assays of human genomic dsDNA employing varying protocols.**

| Sample | Fluorescence on Genexus argon laser @ PMT 32 after 5 min | TAF at 5 min | % of difference relative to perfect match TAF | TAF at 15 min | % of difference relative to perfect match TAF |
| --- | --- | --- | --- | --- | --- |
| 1) YOYO-1 (500 nM) | 0 |  |  |  |  |
| 2) 3849+10kbC->T-WT25C (3.2 pmole/80 ul) | 20055 |  |  |  |  |
| 3) 3849+10kbC->T-MUT25C (3.2 pmole/80 ul) | 20399 |  |  |  |  |
| 4) wt gDNA (2 ng/80 ul) | 7091 |  |  |  |  |
| 5) wt gDNA (2 ng/80 ul) + 3849+10kbC->T-WT25C (perfect) * | 30228 | 10173 |  | 10115 |  |
| 6) wt gDNA (2 ng/80 ul) + 3849+10kbC->T-MUT25C (1bp A-C) * | 23481 | 3082 | - 70 | 1614 | - 84 |
| 7) 3849+10kbC->T-WT25C (3.2 pmole/77.9 ul)** | 22696 |  |  |  |  |
| 8) 3849+10kbC->T-MUT25C (3.2 pmole/77.9 ul)** | 20253 |  |  |  |  |
| 9) wt gDNA (2 ng/80 ul) + 3849+10kbC->T-WT25C (perfect)** |  |  |  | 10264 |  |
| 10) wt gDNA (2ng/80ul) + 3849+10kbC->T-MUT25C (1bp A-C)** |  |  |  | 5218 | - 49 |

| Sample | TAF at 20 min | % of difference relative to perfect match TAF | TAF at 25 min | % of difference relative to perfect match TAF | TAF at 30 min | % of difference relative to perfect match TAF |
| --- | --- | --- | --- | --- | --- | --- |
| 5) wt gDNA (2 ng/80 ul) + 3849+10kbC->T-WT25C (perfect) * | 10279 |  | 10335 |  | 10503 |  |
| 6) wt gDNA (2 ng/80 ul) + 3849+10kbC->T-MUT25C (1bp A-C) * | 977 | - 90 | 603 | - 94 | 113 | - 99 |
| 9) wt gDNA (2 ng/80 ul) + 3849+10kbC->T-WT25C (perfect)** | 10458 |  | 10492 |  | 10476 |  |
| 10) wt gDNA (2ng/80ul) + 3849+10kbC->T-MUT25C (1bp A-C)** | 4662 | - 55 | 3944 | - 62 | 3546 | - 66 |

**Table S10.** Continued

| Sample | TAF at 35 min | % of difference relative to perfect match TAF | TAF at 40 min | % of difference relative to perfect match TAF | TAF at 45 min | % of difference relative to perfect match TAF |
| --- | --- | --- | --- | --- | --- | --- |
| 5) wt gDNA (2 ng/80 ul) + 3849+10kbC->T-WT25C (perfect) * | 10625 |  | 10737 |  | 10847 |  |
| 6) wt gDNA (2 ng/80 ul) + 3849+10kbC->T-MUT25C (1bp A-C) * | 227 | - 98 | < 0 | - 100 | < 0 | - 100 |
| 9) wt gDNA (2 ng/80 ul) + 3849+10kbC->T-WT25C (perfect)** | 10233 |  | 10778 |  | 10551 |  |
| 10) wt gDNA (2ng/80ul) + 3849+10kbC->T-MUT25C (1bp A-C)** | 3216 | - 69 | 3025 | - 72 | 2833 | - 73 |

| Sample | TAF at 50 min | % of difference relative to perfect match TAF | TAF at 55 min | % of difference relative to perfect match TAF | TAF at 60 min | % of difference relative to perfect match TAF |
| --- | --- | --- | --- | --- | --- | --- |
| 5) wt gDNA (2 ng/80 ul) + 3849+10kbC->T-WT25C (perfect) * | 10945 |  | 10872 |  | 10695 |  |
| 6) wt gDNA (2 ng/80 ul) + 3849+10kbC->T-MUT25C (1bp A-C) * | < 0 | - 100 | < 0 | - 100 | < 0 | - 100 |
| 9) wt gDNA (2 ng/80 ul) + 3849+10kbC->T-WT25C (perfect)** | 10973 |  | 11699 |  | 12112 |  |
| 10) wt gDNA (2ng/80ul) + 3849+10kbC->T-MUT25C (1bp A-C)** | 2592 | - 76 | 1788 | - 85 | 1499 | - 88 |

The target was human genomic dsDNA, wild-type for *CFTR*. The 25-mer probes were 3849+10kbC->T-WT25C (wild-type) and 3849+10kbC->T-MUT25C (mutant). 500 nM YOYO-1 and 40 mM TMA-Cl were present in each sample. TAF indicates Triplex-Associated Fluorescence.

* Probe and gDNA were mixed, then YOYO-1 was added. Reaction mixtures were incubated for 5 min and then irradiated.

** Probe and YOYO-1 were mixed, incubated for 5 min and irradiated. Then gDNA was added and the reaction mixture was incubated for 5 min and irradiated.
