# Supplementary material for: Heteropolymeric Triplex-Based Genomic Assay® to Detect Pathogens or Single-Nucleotide Polymorphisms in Human Genomic Samples
Source: PLoS One. 2007 Mar 21;2(3):e305. doi: 10.1371/journal.pone.0000305 (PMC1810429; doi:10.1371/journal.pone.0000305)
Supplement: Table S11. — Assays of human genomic dsDNA for CFTR 3849+10kbC→T (1 bp A–C mismatch) in the presence of 600 nM YOYO-1 and 40 mM TMA-Cl. The emission values giving rise to Supplementary Figure S5 data are shown. The specificity of the triplex assay in detecting CFTR 3849+10kbC→T in mismatched triplexes is demonstrated in reaction mixtures containing 600 nM YOYO-1 and 40 mM TMA-Cl. (0.05 MB DOC) [file pone.0000305.s017.doc]

**Table S11. Assays of human genomic dsDNA for *CFTR* 3849+10kbC->T (1 bp A-C mismatch) in the presence of 600 nM YOYO-1 and 40 mM TMA-Cl.**

| Sample | Fluorescence on Genexus argon laser @ PMT 32 after 5 min | TAF | % of difference relative to perfect match TAF | Fluorescence on Genexus argon laser @ PMT 32 after 15 min | TAF | % of difference relative to perfect match TAF |
| --- | --- | --- | --- | --- | --- | --- |
| 1) YOYO-1 (600 nM) | 0 |  |  | 0 |  |  |
| 2) 3849+10kbC->T-WT25C (3.2 pmole) (antisense) | 19357 |  |  | 18605 |  |  |
| 3) 3849+10kbC->T-MUT25C (3.2 pmole) (antisense) | 15770 |  |  | 14789 |  |  |
| 4) wt gDNA (2 ng) | 4569 |  |  | 4489 |  |  |
| 5) wt gDNA (2 ng) + 3849+10kbC->T-WT25C (perfect) | 25537 | 6180 |  | 24491 | 5886 |  |
| 6) wt gDNA (2 ng) + 3849+10kbC->T-MUT25C (1bp A-C) | 20005 | 4235 | - 31 | 19325 | 4536 | - 23 |

| Sample | Fluorescence on Genexus argon laser @ PMT 32 after 25 min | TAF | % of difference relative to perfect match TAF | Fluorescence on Genexus argon laser @ PMT 32 after 35 min | TAF | % of difference relative to perfect match TAF |
| --- | --- | --- | --- | --- | --- | --- |
| 1) YOYO-1 (600 nM) | 0 |  |  | 0 |  |  |
| 2) 3849+10kbC->T-WT25C (3.2 pmole) (antisense) | 17736 |  |  | 16990 |  |  |
| 3) 3849+10kbC->T-MUT25C (3.2 pmole) (antisense) | 14057 |  |  | 13345 |  |  |
| 4) wt gDNA (2 ng) | 4471 |  |  | 4431 |  |  |
| 5) wt gDNA (2 ng) + 3849+10kbC->T-WT25C (perfect) | 23790 | 6054 |  | 22937 | 5947 |  |
| 6) wt gDNA (2 ng) + 3849+10kbC->T-MUT25C (1bp A-C) | 18845 | 4788 | - 21 | 18073 | 4728 | - 20 |

**Table S11.** Continued

| Sample | Fluorescence on Genexus argon laser @ PMT 32 after 45 min | TAF | % of difference relative to perfect match TAF | Fluorescence on Genexus argon laser @ PMT 32 after 55 min | TAF | % of difference relative to perfect match TAF |
| --- | --- | --- | --- | --- | --- | --- |
| 1) YOYO-1 (600 nM) | 0 |  |  | 0 |  |  |
| 2) 3849+10kbC->T-WT25C (3.2 pmole) (antisense) | 16460 |  |  | 15883 |  |  |
| 3) 3849+10kbC->T-MUT25C (3.2 pmole) (antisense) | 12816 |  |  | 12395 |  |  |
| 4) wt gDNA (2 ng) | 4348 |  |  | 4300 |  |  |
| 5) wt gDNA (2 ng) + 3849+10kbC->T-WT25C (perfect) | 22295 | 5835 |  | 21859 | 5976 |  |
| 6) wt gDNA (2 ng) + 3849+10kbC->T-MUT25C (1bp A-C) | 17530 | 4714 | - 19 | 16998 | 4603 | - 23 |

| Sample | Fluorescence on Genexus argon laser @ PMT 32 after 65 min | TAF | % of difference relative to perfect match TAF | Fluorescence on Genexus argon laser @ PMT 32 after 96 hr | TAF | % of difference relative to perfect match TAF |
| --- | --- | --- | --- | --- | --- | --- |
| 1) YOYO-1 (600 nM) | 0 |  |  | 0 |  |  |
| 2) 3849+10kbC->T-WT25C (3.2 pmole) (antisense) | 15510 |  |  | 11841 |  |  |
| 3) 3849+10kbC->T-MUT25C (3.2 pmole) (antisense) | 12138 |  |  | 11118 |  |  |
| 4) wt gDNA (2 ng) | 4267 |  |  | 5037 |  |  |
| 5) wt gDNA (2 ng) + 3849+10kbC->T-WT25C (perfect) | 21462 | 5952 |  | 20091 | 8250 |  |
| 6) wt gDNA (2 ng) + 3849+10kbC->T-MUT25C (1bp A-C) | 16634 | 4496 | - 24 | 16116 | 4998 | - 39 |

The target was human genomic dsDNA, wild-type for *CFTR* 3849+10kbC->T. The 25-mer probes were 3849+10kbC->T-WT25C (wild-type) and 3849+10kbC->T-MUT25C (mutant). 600 nM YOYO-1 and 40 mM TMA-Cl were present in each sample. TAF indicates Triplex-Associated Fluorescence.
