# Supplementary material for: Heteropolymeric Triplex-Based Genomic Assay® to Detect Pathogens or Single-Nucleotide Polymorphisms in Human Genomic Samples
Source: PLoS One. 2007 Mar 21;2(3):e305. doi: 10.1371/journal.pone.0000305 (PMC1810429; doi:10.1371/journal.pone.0000305)
Supplement: Table S12. — Assays of human genomic dsDNA for CFTR 3849+10kbC→T (1 bp A–C mismatch) in the presence of 500 nM YOYO-1 and 45 mM TMA-Cl. The emission values giving rise to Supplementary Figure S6 data are shown. The specificity of the triplex assay in detecting CFTR 3849+10kbC→T in mismatched triplexes is demonstrated in reaction mixtures containing 500 nM YOYO-1 and 45 mM TMA-Cl. (0.05 MB DOC) [file pone.0000305.s018.doc]

**Table S12. Assays of human genomic dsDNA for *CFTR* 3849+10kbC->T (1 bp A-C mismatch) in the presence of 500 nM YOYO-1 and 45 mM TMA-Cl.**

| Sample | Fluorescence on Genexus argon laser @ PMT 32 after 5 min | TAF | % of difference relative to perfect match TAF | Fluorescence on Genexus argon laser @ PMT 32 after 15 min | TAF | % of difference relative to perfect match TAF |
| --- | --- | --- | --- | --- | --- | --- |
| 1) YOYO-1 (500 nM) | 0 |  |  | 0 |  |  |
| 2) 3849+10kbC->T-WT25C (3.2 pmole) (antisense) | 17843 |  |  | 17324 |  |  |
| 3) 3849+10kbC->T-MUT25C (3.2 pmole) (antisense) | 20814 |  |  | 19859 |  |  |
| 4) wt gDNA (2 ng) | 4901 |  |  | 4883 |  |  |
| 5) wt gDNA (2 ng) + 3849+10kbC->T-WT25C (perfect) | 30844 | 13001 |  | 30250 | 12926 |  |
| 6) wt gDNA (2 ng) + 3849+10kbC->T-MUT25C (1bp A-C) | 23794 | 2980 | - 77 | 23095 | 3237 | - 75 |

| Sample | Fluorescence on Genexus argon laser @ PMT 32 after 25 min | TAF | % of difference relative to perfect match TAF | Fluorescence on Genexus argon laser @ PMT 32 after 35 min | TAF | % of difference relative to perfect match TAF |
| --- | --- | --- | --- | --- | --- | --- |
| 1) YOYO-1 (500 nM) | 0 |  |  | 0 |  |  |
| 2) 3849+10kbC->T-WT25C (3.2 pmole) (antisense) | 17110 |  |  | 16884 |  |  |
| 3) 3849+10kbC->T-MUT25C (3.2 pmole) (antisense) | 19746 |  |  | 19378 |  |  |
| 4) wt gDNA (2 ng) | 4793 |  |  | 4769 |  |  |
| 5) wt gDNA (2 ng) + 3849+10kbC->T-WT25C (perfect) | 29793 | 12683 |  | 29383 | 12499 |  |
| 6) wt gDNA (2 ng) + 3849+10kbC->T-MUT25C (1bp A-C) | 22804 | 3058 | - 76 | 22368 | 2990 | - 76 |

**Table S12.** Continued

| Sample | Fluorescence on Genexus argon laser @ PMT 32 after 45 min | TAF | % of difference relative to perfect match TAF | Fluorescence on Genexus argon laser @ PMT 32 after 55 min | TAF | % of difference relative to perfect match TAF |
| --- | --- | --- | --- | --- | --- | --- |
| 1) YOYO-1 (500 nM) | 0 |  |  | 0 |  |  |
| 2) 3849+10kbC->T-WT25C (3.2 pmole) (antisense) | 16541 |  |  | 16326 |  |  |
| 3) 3849+10kbC->T-MUT25C (3.2 pmole) (antisense) | 19064 |  |  | 18757 |  |  |
| 4) wt gDNA (2 ng) | 4706 |  |  | 4669 |  |  |
| 5) wt gDNA (2 ng) + 3849+10kbC->T-WT25C (perfect) | 28925 | 12384 |  | 28506 | 12180 |  |
| 6) wt gDNA (2 ng) + 3849+10kbC->T-MUT25C (1bp A-C) | 22066 | 3002 | - 76 | 21606 | 2849 | - 77 |

| Sample | Fluorescence on Genexus argon laser @ PMT 32 after 65 min | TAF | % of difference relative to perfect match TAF | Fluorescence on Genexus argon laser @ PMT 32 after 24 hr | TAF | % of difference relative to perfect match TAF |
| --- | --- | --- | --- | --- | --- | --- |
| 1) YOYO-1 (500 nM) | 0 |  |  | 0 |  |  |
| 2) 3849+10kbC->T-WT25C (3.2 pmole) (antisense) | 16160 |  |  | 17207 |  |  |
| 3) 3849+10kbC->T-MUT25C (3.2 pmole) (antisense) | 18623 |  |  | 22473 |  |  |
| 4) wt gDNA (2 ng) | 4557 |  |  | 4697 |  |  |
| 5) wt gDNA (2 ng) + 3849+10kbC->T-WT25C (perfect) | 28328 | 12168 |  | 29230 | 12023 |  |
| 6) wt gDNA (2 ng) + 3849+10kbC->T-MUT25C (1bp A-C) | 21389 | 2766 | - 77 | 20771 | < 0 | - 100 |

The target was human genomic dsDNA, wild-type for *CFTR* 3849+10kbC->T. The 25-mer probes were 3849+10kbC->T-WT25C (wild-type) and 3849+10kbC->T-MUT25C (mutant). 500 nM YOYO-1 and 45 mM TMA-Cl were present in each sample. TAF indicates Triplex-Associated Fluorescence.
